# Csk-mediated Src family kinase regulation dampens neutrophil infiltration during pulmonary infection

**DOI:** 10.1172/jci.insight.188323

**Published:** 2025-06-10

**Authors:** Wida Amini, Lena Schemmelmann, Jan-Niklas Heming, Marina Oguama, Katharina Thomas, Helena Block, Pia Lindental, Bernadette Bardel, Andreas Margraf, Oliver Soehnlein, Anika Cappenberg, Alexander Zarbock

**Affiliations:** 1 Department of Anesthesiology, Intensive Care and Pain Medicine, and; 2 Department of Cardiothoracic Surgery, University Hospital Muenster, Muenster, Germany.; 3 Institute of Experimental Pathology (ExPat), Center of Molecular Biology of Inflammation (ZMBE), University of Muenster, Muenster, Germany.

**Keywords:** Immunology, Inflammation, Integrins, Neutrophils

## Abstract

Neutrophil recruitment is crucial for pathogen elimination. However, precise control of the inflammatory response prevents overshooting reactions. Neutrophil activation initiates signaling, resulting in integrin β2 (Itgb2) activation and neutrophil arrest. Src family kinases are involved in multiple cellular processes and are negatively regulated by the C-terminal Src kinase (Csk). During this study, we investigated the mechanism by which Csk regulates integrin activation and neutrophil recruitment. Here, we demonstrated that Csk deficiency in murine neutrophils resulted in increased neutrophil adhesion to the endothelium along with decreased neutrophil transmigration into inflamed tissues compared with their littermate controls. In bacterial pneumonia, infected *Csk*-deficient mice showed higher bacterial burdens and decreased neutrophil recruitment, while other immune cell counts and cytokine levels were not significantly different compared to control. Analyses of *Csk*-deficient neutrophils revealed an increased Itgb2 affinity, leading to reduced migration and intravascular crawling. Mechanistically, elevated cAMP levels increased protein kinase A activity, which subsequently enhanced Csk activation. Csk, in turn, suppressed Src family kinase activation through phosphorylation (Y529). Hence, Csk-mediated regulation of neutrophil infiltration contributes to maintain a balanced immune response during bacterial pneumonia.

## Abstract 2

Excessive neutrophil recruitment during bacterial infections can cause organ damage. The C-terminal protein kinase Csk regulates the activation of Src family kinases and thereby inhibits neutrophil recruitment into infected lungs.

## Graphical abstract

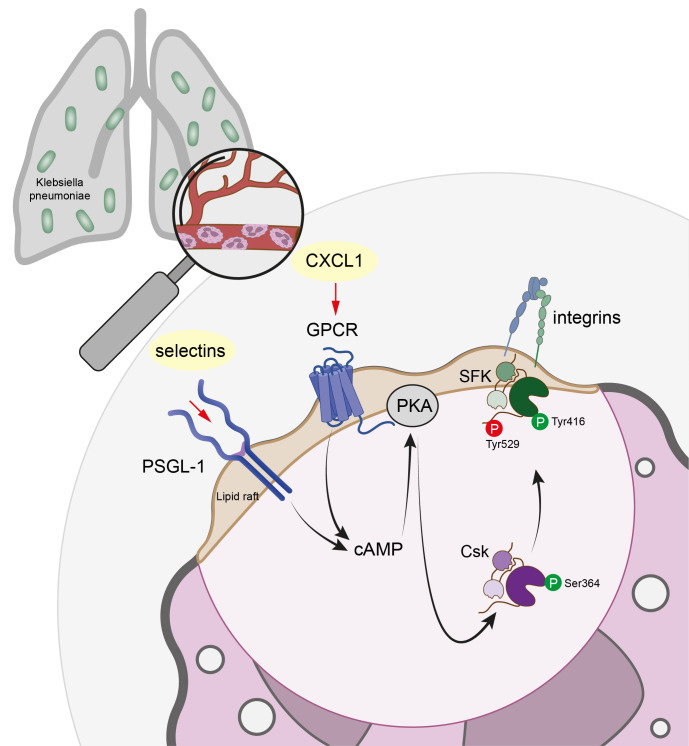

## Introduction

In case of tissue injury or infection, prompt recruitment of neutrophils from the blood stream into the inflamed tissue is crucial for efficient clearance of pathogens, whereas an overwhelming neutrophil activation and recruitment can lead to tissue damage (1–5). The recruitment of neutrophils includes the following steps: capturing, rolling and slow rolling, arrest and subsequent adhesion strengthening, crawling, and transendothelial migration (6,7). The initial phase of the neutrophil recruitment cascade requires contact between neutrophils and the inflamed endothelium. Binding of selectins (E-, L-, and P-selectin) to their respective counter-receptor P-selectin glycoprotein ligand-1 (PSGL-1) on neutrophils induces downstream signaling (6, 8–11). Src family kinases (SFKs) are involved in these processes by mediating slow rolling triggered by PSGL-1 engagement (12–17). The subsequent downstream signaling leads to integrin activation (10, 11). Two pivotal integrins on the surface of neutrophils are lymphocyte-function-associated antigen-1 (LFA-1; CD11a) and macrophage antigen-1 (Mac-1; CD11b) (18–21). Integrin activation can be triggered by selectin-mediated signaling. After selectin engagement, integrins undergo a transition to an intermediate-affinity state (22). SFKs are additionally known to mediate integrin β2–induced (Itgb2-induced) outside-in signaling in neutrophils (12–17). Binding of proinflammatory chemokines to their respective GPCR activates integrins further and leads to a high-affinity conformation (23). Binding of CD11a on neutrophils to its ligand ICAM-1 on the inflamed endothelium is necessary for slow rolling and results in neutrophil arrest and adhesion (10, 24, 25). After adhesion, neutrophils crawl along the endothelium, actively seeking a specific site for transmigration into the tissue. This crawling process is strongly dependent on CD11b (26, 27). Neutrophil recruitment culminates in transmigration from the vasculature into the tissue across the endothelial cell layer along a chemokine gradient (7).

Neutrophils are highly effective cells in combating microbes (28). Their effector functions are essential for the physiological defense against pathogens. During phagocytosis, neutrophils engulf microbes by forming an intracellular phagosome, where pathogens get digested (29). Moreover, activated neutrophils release large amounts of reactive oxygen species (ROS). ROS are critical components of the antimicrobial host defense (20), directly causing oxidative damage to microbial cellular components. Intracellular ROS release supports decondensation of chromatin as prerequisite for the formation of neutrophil extracellular traps (NETs), a network of DNA and proteins (30). NETs allow to capture and kill pathogens (31). A deficiency in neutrophil effector functions can lead to recurring or worsened infections, whereas their upregulation can cause pathologic damage due to chronic inflammation (32).

SFKs are involved in the modulation of neutrophil motility and their adhesion within the vasculature (33). The non–receptor tyrosine kinases play crucial roles in different signaling pathways, thereby regulating neutrophil functions (34, 35). Their activity status is controlled by C-terminal Src kinase (Csk), their most crucial endogenous negative regulator (36, 37). A landmark study by Thomas et al. showed that *Csk*-deficient granulocytes are hyper-responsive, leading to increased adhesion and impaired migration in vitro (38). They used *Csk*-GEcre mice: *Csk*-floxed mice crossed with mice expressing Cre recombinase with a promoter in the granulocyte elastase locus (39). Twenty-four hours after induction of sterile peritonitis with thioglycollate, the number of recruited granulocytes into the peritoneal cavity was reduced in *Csk*-GEcre mice in comparison with littermate controls (38).

The activation level of SFKs is regulated by phosphorylation of 2 regulatory C-terminal tyrosine sites (40, 41). Activation requires phosphorylation of tyrosine 416 (Y416) of the kinase domain (42). Phosphorylation of Y416 induces an activation loop and leads to a conformational change that exposes the catalytic site and enhances substrate binding and enzymatic activity (43). In contrast, phosphorylation of tyrosine 529 (Y529) strengthens the formation of an autoinhibited conformation through stabilization of intramolecular interactions (44–48). The inactivating phosphorylation at Y529 is catalyzed by Csk (44, 49) and inhibits SFK activity (47, 50). Moreover, protein kinase A (PKA) phosphorylates Csk at serine 364 (Ser364) and increases its kinase activity (51). PKA itself is activated by elevated levels of cAMP (52–54).

Each step of the neutrophil recruitment cascade involves precise spatial and temporal interactions among adhesion receptors, stimulants, and intracellular signaling pathways. These processes are tightly regulated. Defects within the signal transduction can lead to reduced adhesion and recruitment of neutrophils in vivo (17, 55). This study aims to explore the intricate interplay between Csk and key components of integrin activation and neutrophil recruitment and focuses on intracellular signaling pathways. Specifically, our research investigates the role of Csk in selectin-, chemokine- and integrin-mediated signaling as well as neutrophil recruitment into inflamed tissues.

## Results

### Csk is required for neutrophil recruitment and bacterial containment in the lungs during Klebsiella pneumoniae–induced pneumonia.

**Figure 1:**
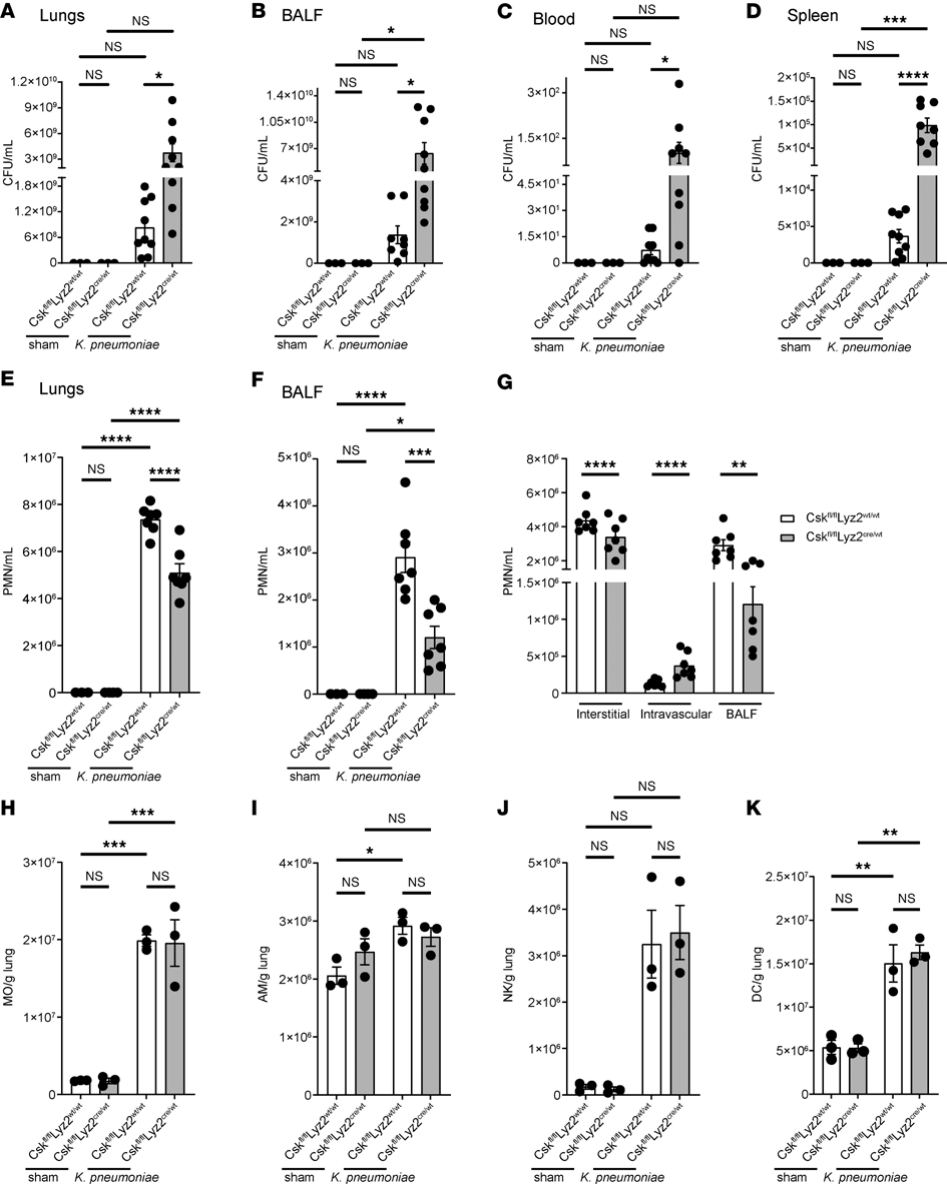
Csk is crucial for neutrophil recruitment and bacterial clearance in *K*. *pneumoniae*–induced pneumonia. (**A**–**K**) *Csk^fl/fl^Lyz2^wt/wt^* and *Csk^fl/fl^Lyz2^cre/wt^* mice were subjected to *K*. *pneumoniae* intratracheal injection or sham surgery. After 24 hours, bacterial burden regarding the colony forming units (CFUs) in lung (**A**), bronchoalveolar fluid (BALF) (**B**), blood (**C**), and spleen (**D**) and neutrophil recruitment into the lungs (**E**) and BALF (**F**) were determined. (**G**) Neutrophil recruitment (CD45^+^Gr-1^+^LyB.2^+^) in *Csk^fl/fl^Lyz2^wt/wt^* and *Csk^fl/fl^Lyz2^cre/wt^* mice in the interstitial, intravascular, and BALF compartments following intratracheal instillation of *K*. *pneumoniae*. Cell count of monocytes (MO; CD45^+^CD11b^+^CX3CR1^+^Ly6C^hi^Ly6G^–^Gr-1^–^) (**H**), alveolar macrophages (AM; CD45^+^CD64^+^F4/80^+^MARCO^+^SiglecF^hi^) (**I**), natural killer cells (NK; CD45^+^CD27^+^CD335^+^) (**J**), and dendritic cells (DC; CD45^+^CD27^–^CD24^+^CD11c^+^MHCII^+^) (**K**) in lung tissue 24 hours after lung infection. *n* as indicated, mean ± SEM. *P < 0.05; **P < 0.01; ***P < 0.001; ****P < 0.0001 by 1-way-ANOVA by Holm-Šídák multiple-comparison test (**A**–**D**), 1-way ANOVA with Tukey’s multiple-comparison test (**E**, **F**, and **H**–**K**), or 2-tailed Student’s *t* test (**G**).

We utilized a murine model of bacterial pneumonia by injecting a pathogenic dose of *Klebsiella* *pneumoniae* intratracheally to investigate whether Csk is involved in immune cell recruitment and the clearance of bacteria in vivo. *K*. *pneumoniae* is a Gram-negative and encapsulated bacterium and a common cause of bacterial pneumonia (56, 57). Twenty-four hours after infection, the bacterial burden was determined by colony forming units (CFUs) in lung tissue, bronchoalveolar fluid (BALF), blood, and spleen. The bacterial burden was significantly higher in all the organs investigated of *Csk^fl/fl^Lyz2^cre/wt^* mice in comparison with the respective control (Figure 1, A–D). In sham-operated mice, no CFUs were detected. Twenty-four hours after lung infection, neutrophil recruitment into the lungs and the alveoli (BALF) was significantly decreased in *Csk^fl/fl^Lyz2^cre/wt^* mice compared with littermate controls (Figure 1, E and F). To further investigate these recruitment changes and differentiate between neutrophils in the interstitial or intravascular compartment, fluorophore-labeled Gr-1 (clone RB6-8C5) was injected intravenously, and harvested lungs were incubated in unlabeled Gr-1. Pulmonary neutrophils were considered fluorophore-negative, intravascular neutrophils fluorophore-positive. This additional analysis unveiled an altered distribution of *Csk*-deficient neutrophils in the lungs (Figure 1G). Fewer neutrophils were recruited to the interstitial compartment, while the number of intravascular neutrophils increased. Subsequently, fewer neutrophils reached the BALF in comparison with the control group (Figure 1G). To address the specificity of the *LysM* promoter, we also investigated whether the recruitment of other immune cells involved in pneumonia are affected. We did not detect any changes in the number of monocytes, alveolar macrophages, natural killer cells, or dendritic cells between control and *Csk*-deficient mice 24 hours after sham surgery or lung infection (Figure 1, H–K, and Supplemental Figure 1, A–D; supplemental material available online with this article; https://doi.org/10.1172/jci.insight.188323DS1). Untreated *Csk^fl/fl^Lyz2^wt/wt^* and *Csk^fl/fl^Lyz2^cre/wt^* mice showed no significant differences in the number of neutrophils and the above-mentioned immune cell types in the lungs or BALF (Supplemental Figure 1, E–N).

Thus, the white blood cell, neutrophil, and monocyte counts in the bone marrow or blood did not differ between the 2 compared groups under baseline conditions or 24 hours after lung infection (Supplemental Figure 2, A–C). Moreover, control and *Csk^fl/fl^Lyz2^cre/wt^* neutrophils showed no significant differences in the surface expression of CD11a, CD11b, CD44, CD62L, CD162, or CXCR2, as determined by FACS analysis (Supplemental Figure 2D). Western blot analysis revealed a significant reduction in Csk expression in neutrophils, whereas no significant changes were detected in monocytes (Supplemental Figure 2, E–H). The *Lyz2* promotor has been demonstrated to partially affect lung epithelial cells (58). This study failed to identify significant differences in the expression of Csk in lung epithelial cells between control and *Csk*-deficient mice, determined by qPCR (Supplemental Figure 2I). These findings indicate that Csk is pivotal for neutrophil recruitment, particularly neutrophil transmigration, and bacterial containment during *K*. *pneumoniae*–induced lung infection.

### Csk regulates neutrophil recruitment during pulmonary infection, up to 36 hours after infection with K. pneumoniae, and after 24 hours during Staphylococcus aureus infection.

To investigate whether Csk modulates immune cell recruitment at later time points, we sacrificed mice 36 hours after intratracheal injection of *K*. *pneumoniae* (Supplemental Figure 3, A–N). The observed differences between *Csk^fl/fl^Lyz2^cre/wt^* mice and littermate controls were also evident after prolonged infection. In general, in comparison with the results obtained 24 hours after lung infection, the bacterial burden was even higher 36 hours after infection (Figure 1, A–D, and Supplemental Figure 3, A–D). The CFUs in lungs, BALF, blood, and spleen were significantly higher and fewer neutrophils were recruited into the lungs and BALF of *Csk*-deficient mice compared with control mice (Supplemental Figure 3, E–F). The recruitment of other immune cells to the lung tissue and BALF did not vary between the compared groups (Supplemental Figure 3, G–N). Survival after 24 hours and 36 hours did not differ between control and *Csk*-deficient mice (data not shown).

**Figure 2:**
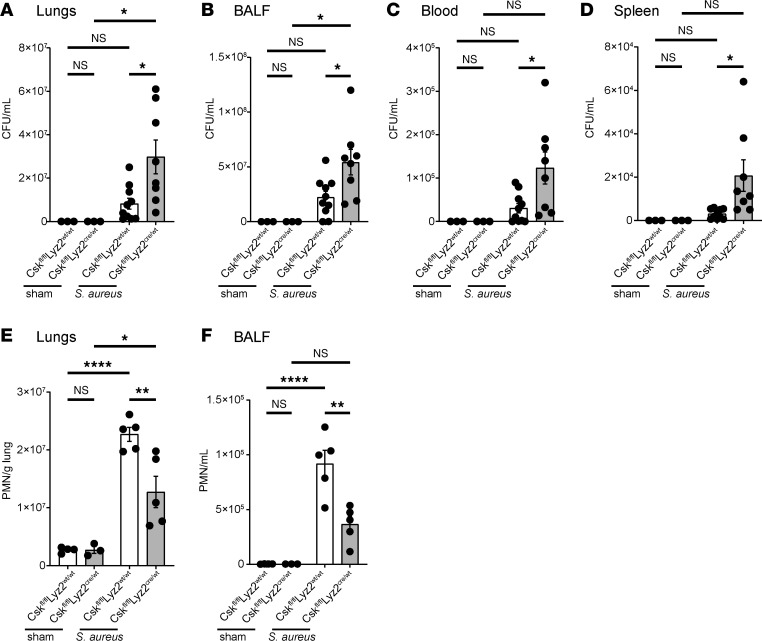
Csk is crucial for neutrophil recruitment and bacterial clearance in *S*. *aureus*–induced pneumonia. (**A**–**F**) *Csk^fl/fl^Lyz2^wt/wt^* and *Csk^fl/fl^Lyz2^cre/wt^* mice were subjected to *S*. *aureus* intratracheal injection or sham surgery. Bacterial burden regarding the colony forming units (CFUs) in lungs (**A**), bronchoalveolar fluid (BALF) (**B**), blood (**C**), and spleen (**D**) and neutrophil recruitment into the lungs (**E**) and BALF (**F**) were determined 24 hours after *S*. *aureus* injection. *n* = 3–10 mice per genotype, mean ± SEM. *P < 0.05; **P < 0.01; ****P < 0.0001 by 1-way ANOVA with Tukey’s multiple-comparison test.

To check whether our findings can be extrapolated to other pathogens, we performed *Staphylococcus aureus*–induced lung infections. *S*. *aureus* is a Gram-positive bacterium and a common cause of hospital-acquired upper respiratory infections, causing a high mortality (58–60). Similar to *K*. *pneumoniae*–infected mice, the CFUs in lungs, BALF, blood, and spleen were significantly higher in *Csk*-deficient mice (Figure 2, A–D) compared with the respective control. In infected *Csk*-deficient mice, higher CFUs were accompanied with a significant reduction in neutrophil recruitment to the lungs and BALF (Figure 2, E and F).

To investigate whether differences in cytokine and chemokine levels between *Csk*-deficient mice and control mice may cause altered recruitment of neutrophils, we performed LEGENDplex analysis of the cytokines and chemokines IL-1α, IL-1β, IL-6, IL-10, IL-17A, IL-23, IL-27, MCP-1, and TNF in the lungs (Supplemental Figure 4, A–I), BALF (Supplemental Figure 5, A–I), and serum (Supplemental Figure 6, A–I) of sham-operated and *K*. *pneumoniae* 24-hour–infected mice. No discernible differences were observed between control and *Csk*-deficient mice under steady-state conditions. Following infection, an increase in most of the investigated proteins was observed, particularly in lung and BALF samples. However, *Csk* deficiency did not cause altered cytokine or chemokine levels. Since the *Lyz2* promoter can also affect other cells besides neutrophils (61), we used reconstitution experiments to analyze whether the observed phenotype can be exclusively attributed to neutrophils. Neutropenic *Mcl-1^fl/fl^Ly6G* mice received a 24-hour lung infection with *K*. *pneumoniae* and a simultaneous intravenous reconstitution with isolated and viable *Csk*-deficient or control neutrophils (Supplemental Figure 7, A–F). Again, we observed a significant decrease in the number of *Csk*-deficient neutrophils recruited to the lungs and the BALF (Supplemental Figure 7, A and B). The CFUs in the lungs and blood exhibited analogous tendencies, while those in the spleen and BALF demonstrated a significant increase after reconstitution with *Csk*-deficient neutrophils (Supplemental Figure 7, C–F). Our in vivo data demonstrate that Csk is essential for neutrophil recruitment in the immune response following infection with *K*. *pneumoniae* as well as *S*. *aureus*, representing Gram-negative as well as Gram-positive pathogens.

### Neutrophil effector functions do not depend on Csk.

**Figure 3:**
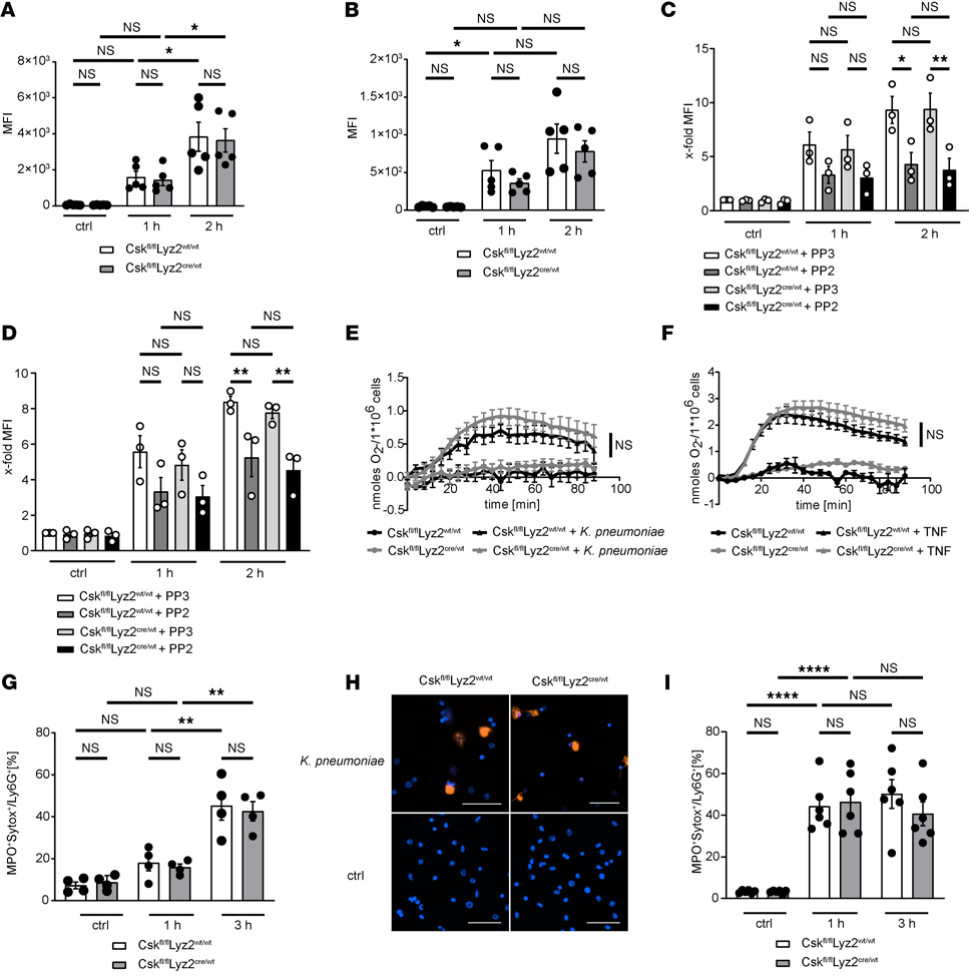
Csk is not involved in the regulation of neutrophil effector functions. (**A** and **B**) Phagocytosis of pHrodo-labeled *K*. *pneumoniae*, opsonized with serum (**A**) or IgG antibody (**B**), by *Csk^fl/fl^Lyz2^wt/wt^* and *Csk^fl/fl^Lyz2^cre/wt^* neutrophils. (**C** and **D**) Phagocytosis of phRodo-labeled *K*. *pneumoniae*, opsonized with serum (**C**) or IgG antibody (**D**), by *Csk^fl/fl^Lyz2^wt/wt^* and *Csk^fl/fl^Lyz2^cre/wt^* neutrophils treated with PP2, an SFK-specific inhibitor, or PP3 as negative control. (**E** and **F**) Adhesion-dependent oxidative burst of *Csk^fl/fl^Lyz2^wt/wt^* and *Csk^fl/fl^Lyz2^cre/wt^* neutrophils plated on fibrinogen alone or in the presence of serum-opsonized *K*. *pneumoniae* (**E**) or TNF (**F**). (**G**) NET formation of *Csk^fl/fl^Lyz2^wt/wt^* and *Csk^fl/fl^Lyz2^cre/wt^* neutrophils upon stimulation with untreated *K*. *pneumoniae* after 1 hour and 3 hours analyzed by flow cytometry. (**H**) Representative images of NET formation of *Csk^fl/fl^Lyz2^wt/wt^* and *Csk^fl/fl^Lyz2^cre/wt^* neutrophils after 3 hours of incubation with *K*. *pneumoniae*. DAPI staining is shown in blue color; H3Cit staining is visualized in orange color. Scale bars: 30 μm. (**I**) NET formation upon 1 hour or 3 hours of PMA stimulation analyzed by flow cytometry. *n* as indicated; *n* = 5 for **E** and **F**, mean ± SEM. *P < 0.05; **P < 0.01; ****P < 0.0001 by 2-way ANOVA with Tukey’s multiple-comparison test (**A**–**D**, **G**, and **I**) or 2-way ANOVA with Bonferroni’s multiple-comparison test (**E** and **F**).

Next, we sought to clarify whether the observed effect of Csk deficiency in the murine pneumonia model is due to changes in neutrophil recruitment or bacterial clearance or both. To this end, we used *K*. *pneumoniae* as stimuli to investigate neutrophil effector functions. First, we performed phagocytosis assays of pHRodo-labeled bacteria. To distinguish between complement-mediated and Fc-mediated phagocytosis, bacteria were opsonized with either mouse serum or mouse IgG. Phagocytosis of serum- and IgG-opsonized *K*. *pneumoniae* was not affected in *Csk*-deficient neutrophils compared to control neutrophils (Figure 3, A and B). It is known that SFKs are involved in phagocytosis (62–64). Therefore, we further investigated the phagocytosis of neutrophils incubated with PP2, a specific inhibitor of SFKs, and PP3, its respective control. Inhibition of SFKs indeed reduced phagocytosis in both pathways, complement-mediated (Figure 3C) and Fc-mediated phagocytosis (Figure 3D). However, PP2 affected knockout and control neutrophils to the same extent.

Superoxide production is another neutrophil effector function required for efficient bacterial clearance (65). Control neutrophils exhibited a significantly increased oxidative burst when plated on fibrinogen in the presence of serum-opsonized *K*. *pneumoniae* compared with fibrinogen alone (Figure 3E). The same effect was apparent in *Csk*-deficient neutrophils. The oxidative burst was slightly, but not significantly, increased in *Csk*-deficient neutrophils compared with respective controls (Figure 3E). WT neutrophils showed no significant oxidative burst in the presence of non-opsonized bacteria (data not shown); therefore, opsonized bacteria were used. Similarly, the oxidative burst in response to a sterile stimulus did not significantly differ between *Csk*-deficient and control neutrophils (Figure 3F).

Next, we analyzed NET formation of control and *Csk*-deficient neutrophils after stimulation with *K*. *pneumoniae* (Figure 3, G and H). We did not observe any significant differences in the release of NETs between *Csk*-deficient neutrophils and control neutrophils. Figure 3H shows exemplary images of unstimulated neutrophils and neutrophils incubated with *K*. *pneumoniae*. NET formation caused by PMA as a sterile stimulus was also tested for FACS analysis, which yielded similar results (Figure 3I). Taken together, our results demonstrate that Csk is not involved in the neutrophil effector functions phagocytosis, ROS production, or NET formation after stimulation with *K*. *pneumoniae* or sterile substances.

### Csk is involved in integrin-mediated neutrophil slow rolling, chemokine-induced arrest and recruitment in vivo.

**Figure 4:**
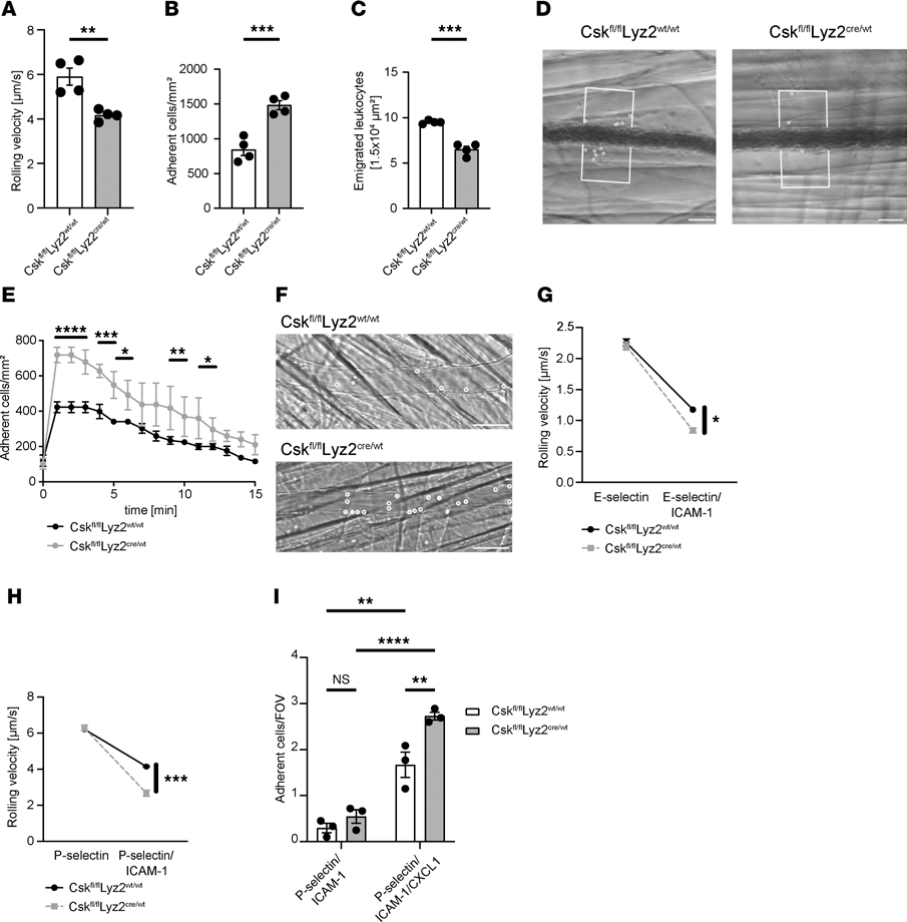
Csk is involved in integrin-mediated neutrophil slow rolling, chemokine-induced arrest, and neutrophil recruitment in vivo. (**A**–**D**) Intravital microscopy of postcapillary venules in the murine cremaster muscle 2 hours after intrascrotal TNF injection. (**A**) Rolling velocities of neutrophils from *Csk^fl/fl^Lyz2^wt/wt^* and *Csk^fl/fl^Lyz2^cre/wt^* mice. (**B**) Adherent cells per square millimeter and (**C**) the number of extravasated cells per 1.5 × 10^4^ μm^2^ tissue area surrounding postcapillary venules. (**D**) Representative reflected light oblique transillumination microscopy photographs. White circles represent transmigrated neutrophils within the tissue, boxes indicate the analyzed tissue area. Scale bars: 50 μm. (**E** and **F**) Chemokine-induced arrest of neutrophils in postcapillary venules of *Csk^fl/fl^Lyz2^wt/wt^* and *Csk^fl/fl^Lyz2^cre/wt^* mice before and following CXCL1 injection. (**E**) Number of adherent cells per mm^2^. (**F**) Representative images of postcapillary venules of *Csk^fl/fl^Lyz2^wt/wt^* and *Csk^fl/fl^Lyz2^cre/wt^* mice following CXCL1 injection. White circles represent adherent neutrophils within the vessel. Scale bars: 50 μm. (**G**–**I**) Carotid cannulas were placed in *Csk^fl/fl^Lyz2^wt/wt^* and *Csk^fl/fl^Lyz2^cre/wt^* mice and connected to autoperfused flow chambers. Average rolling velocity of neutrophils on (**G**) E-selectin and E-selectin/ICAM-1 and (**H**) P-selectin and P-selectin/ICAM-1. (**I**) Number of adherent cells on P-selectin/ICAM-1– and P-selectin/ICAM-1/CXCL1–coated flow chambers. *n* as indicated; *n* = 3 for **E**–**H**, mean ± SEM. *P < 0.05; **P < 0.01; ***P < 0.001; ****P < 0.0001 by 2-tailed Student’s *t* test (**A**–**C**), 2-way-ANOVA with Šídák’s multiple-comparison test (**E**), or 2-way ANOVA with Tukey’s multiple-comparison test (**G**–**I**).

Since neutrophil recruitment in the absence of Csk was affected in the lung infection models (Figure 1, E–G, and Figure 2, E and F), we performed intravital microscopy of the inflamed murine cremaster muscle of *Csk^fl/fl^Lyz2^wt/wt^* and *Csk^fl/fl^Lyz2^cre/wt^* mice following intrascrotal injection of TNF. Jung et al. showed in WT and different genetically modified mice that approximately 90% of all cells in the inflamed cremaster muscle are granulocytes (66). This technique allows the investigation of the different steps of the leukocyte recruitment cascade in detail. Neutrophil rolling velocity on the inflamed endothelium was significantly reduced in *Csk^fl/fl^Lyz2^cre/wt^* mice in comparison with control mice (Figure 4A). The number of adherent cells was significantly higher (Figure 4B), while the number of transmigrated neutrophils was significantly reduced (Figure 4, C and D), suggesting that Csk plays an essential role in the regulation of neutrophil recruitment. To further dissect whether Csk is involved in GPCR-induced neutrophil arrest, we conducted intravital microscopy of the cremaster muscle before and after administering CXCL1 (Figure 4, E and F). Under baseline conditions, the number of adherent neutrophils was comparable between *Csk^fl/fl^Lyz2^cre/wt^* and control mice. Immediately after CXCL1 injection, *Csk^fl/fl^Lyz2^cre/wt^* showed a significantly higher number of adherent neutrophils compared with control mice (Figure 4, E and F). Neutrophil slow rolling is strongly dependent on selectin-mediated integrin activation (6). To investigate the role of Csk in selectin-mediated slow rolling, autoperfused flow chamber experiments were performed. Control neutrophils exhibited a reduced rolling velocity on E-selectin plus ICAM-1 compared with E-selectin alone (Figure 4G). Adding ICAM-1 to P-selectin–coated flow chambers significantly reduced the rolling velocity of control neutrophils (Figure 4H). Notably, *Csk^fl/fl^Lyz2^cre/wt^* neutrophils displayed a significantly more pronounced decrease in the rolling velocities on E-selectin/ICAM-1–coated and P-selectin/ICAM-1–coated flow chambers compared with control neutrophils (Figure 4, G and H). Adding CXCL1 to P-selectin–and ICAM-1–coated flow chambers led to neutrophil arrest and enabled us to analyze chemokine-mediated integrin-dependent neutrophil arrest. Consistent with the results of the chemokine-induced arrest assay in vivo, the autoperfused flow chambers also demonstrated that *Csk^fl/fl^Lyz2^cre/wt^* neutrophils adhered significantly more compared with control neutrophils (Figure 4I). In summary, our results confirmed the involvement of Csk in selectin-induced slow neutrophil rolling and CXCL1-induced neutrophil arrest.

### Csk is required for intravascular crawling and migration of neutrophils.

**Figure 5:**
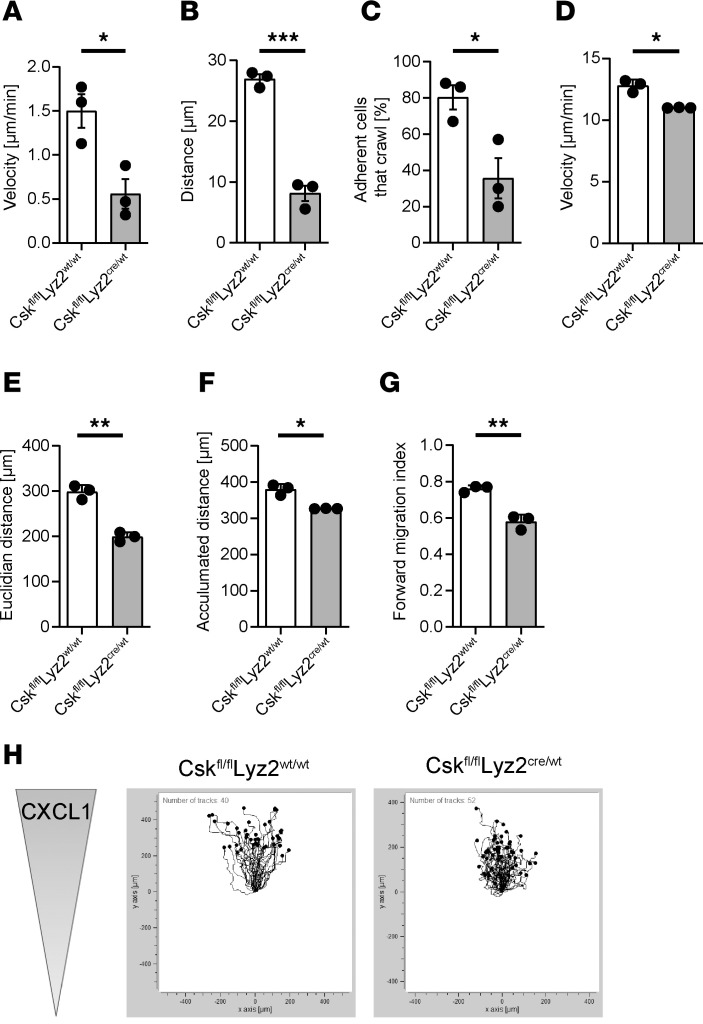
Csk is required for intravascular crawling and migration of leukocytes. (**A**–**C**) Intravascular crawling of leukocytes in venules of the murine cremaster muscle during superfusion with CXCL2. Presented are the mean crawling velocity of adherent cells (**A**), mean distance crawled by adherent cells (**B**), and the percentage of adherent cells that crawled (**C**) in *Csk^fl/fl^Lyz2^wt/wt^* and *Csk^fl/fl^Lyz2^cre/wt^* mice. (**D**–**H**) Chemotaxis of isolated *Csk^fl/fl^Lyz2^wt/wt^* and *Csk^fl/fl^Lyz2^cre/wt^* neutrophils on fibronectin in response to a soluble CXCL1 gradient in vitro. Migration velocity (**D**), Euclidian and accumulated distance (**E** and **F**), and forward migration index (**G**) of chemotaxing neutrophils. (**H**) Representative trajectory plots of *Csk^fl/fl^Lyz2^wt/wt^* and *Csk^fl/fl^Lyz2^cre/wt^* neutrophils. *n* = 3 mice per genotype, mean ± SEM. *P < 0.05; **P < 0.01; ***P < 0.001 by 2-tailed Student’s *t* test.

To assess whether Csk is involved in intravascular crawling, we performed intravital microscopy of the cremaster muscle during CXCL2 superfusion and analyzed neutrophil crawling. The crawling velocity (Figure 5A), crawling distance (Figure 5B), and percentage of neutrophils that crawl (Figure 5C) were significantly reduced in *Csk^fl/fl^Lyz2^cre/wt^* mice compared with control mice. Additionally, we used a chemotactic migration assay to assess the overall migration capability of isolated neutrophils (Figure 5, D–H). Control neutrophils, plated on fibronectin-coated slides, migrated efficiently along a CXCL1 gradient. Conversely, neutrophils lacking Csk showed less effective migration along the chemotactic gradient. The migration velocity (Figure 5D), migration distance (Figure 5, E and F), and forward migration index (Figure 5G) of *Csk^fl/fl^Lyz2^cre/wt^* neutrophils were significantly diminished compared with neutrophils from littermate controls. The altered migration behavior of *Csk*-deficient neutrophils is visualized by trajectory plots (Figure 5H). The process of efficient transmigration of neutrophils into the tissue relies on CD11a-dependent adhesion followed by CD11b-dependent crawling (27). The impaired crawling and migration ability of *Csk*-deficient neutrophils could thus be an indication of disturbed CD11b activity.

### Csk is involved in CD11a and CD11b activity regulation.

**Figure 6:**
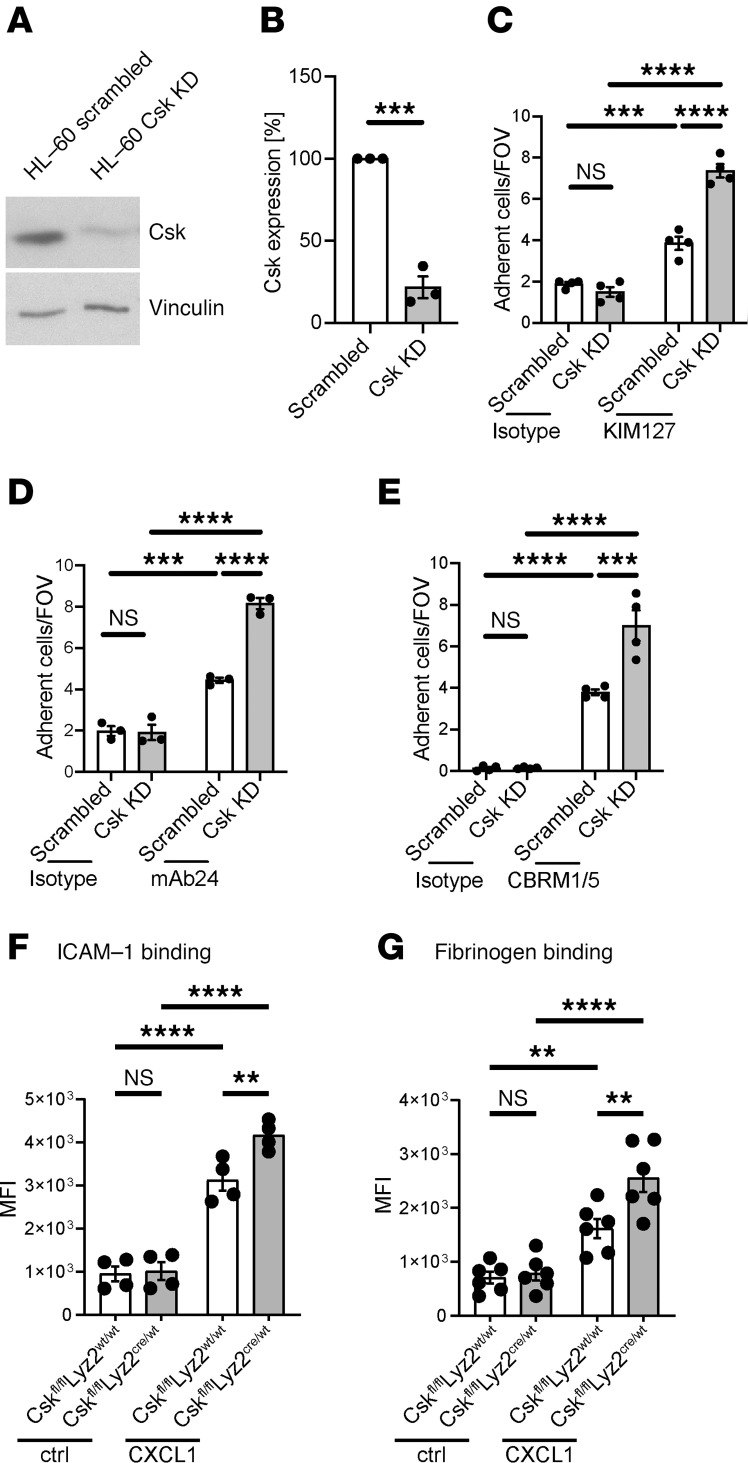
Csk is involved in CD11a and CD11b activation regulation. (**A** and **B**) Csk protein levels in HL-60 cells after lentiviral transduction with scrambled shRNA or shRNA against Csk. (**A**) Representative Western blots of HL-60 scrambled or HL-60 Csk-knockdown lysates, immunoblotted against total Csk (tCsk) and Vinculin. (**B**) Quantification of tCsk levels by Western blot. (**C**–**E**) HL-60 cells were analyzed using a flow chamber adhesion assay with E-selectin and either an Ab specific for the intermediate conformation of CD11a (KIM127) (**C**) or P-selectin, IL-8 and an Ab specific for the full open conformation of CD11a (mAb24) (**D**), or an Ab specific for the activation epitope of CD11b (CBRM1/5) (**E**), or a control IgG Ab. Adherent cells per field of view were counted. Analysis of (**F**) ICAM-1 binding and (**G**) CD11b-dependent fibrinogen binding in unstimulated and CXCL1-stimulated *Csk^fl/fl^Lyz2^wt/wt^* and *Csk^fl/fl^Lyz2^cre/wt^* neutrophils, measured by flow cytometry. *n* as indicated, mean ± SEM. **P < 0.01; ***P < 0.001; ****P < 0.0001 by 2-tailed Student’s *t* test (**B**) or 1-way ANOVA with Tukey’s multiple-comparison test (**C**–**G**).

To validate the impact of Csk on the activation status of neutrophilic integrins, we performed a series of in vitro flow chamber assays using control and Csk-knockdown cells of the human promyelocytic cell line HL-60 (Figure 6, A–E). The knockdown efficiency was confirmed via Western blotting (Figure 6, A and B). Highly specific antibodies targeting the activation epitopes of CD11a and CD11b are available for human integrins, but not for murine. Subsequently, HL-60 cells were utilized in reporter antibody flow chamber experiments (Figure 6, C–E). KIM127 is a mouse monoclonal antibody specific for human CD11a and targets an epitope near the bend of the β2 subunit (67). This epitope is only accessible when CD11a is present in its extended conformation (67–69). In addition, the monoclonal reporter antibody 24 (mAb24) targets an epitope between 2 subunits of the open, high-affinity conformation of CD11a. Therefore, binding of mAb24 is an indicator of the high-affinity state of CD11a (70).

Adhesion of scrambled HL-60 cells was significantly increased on KIM127- or mAb24-coated glass capillaries in comparison with the isotype control (Figure 6, C and D). HL-60 cells deficient in Csk expression exhibited a significantly increased number of adherent cells on KIM127- and mAb24-coated surfaces compared with the scrambled control (Figure 6, C and D). This suggests that Csk may have a significant role in regulating the activity status of CD11a in human cells. Additional flow chamber experiments were conducted to investigate the role of Csk in CD11b activation using the monoclonal antibody CBRM1/5 (Figure 6E). CBRM1/5 targets an activation-specific epitope on CD11b molecules found on neutrophils following stimulation (71). Csk-knockdown HL-60 cells exhibited a higher number of adherent cells on CBRM1/5 compared with scrambled control cells (Figure 6E). Additionally, no difference between scrambled and Csk-knockdown cells was observed in the isotype control in all experiments (Figure 6, C–E). Taken together, these data demonstrate that Csk is involved in the regulation of activation of Itgb2, CD11a, and CD11b in human neutrophils.

To further confirm the function of Csk during chemokine-mediated integrin activation, we examined ICAM-1 binding of isolated murine neutrophils via flow cytometry as a readout of CD11a activation regulation (Figure 6F). Unstimulated control and *Csk^fl/fl^Lyz2^cre/wt^* neutrophils bound equal amounts of ICAM-1 (Figure 6F). After CXCL1 stimulation, *Csk^fl/fl^Lyz2^cre/wt^* neutrophils bound significantly more ICAM-1 compared with stimulated control neutrophils (Figure 6F). To further elucidate the involvement of Csk in CD11b regulation, we conducted a fibrinogen binding assay, as fibrinogen binding is predominantly dependent on CD11b (Figure 6G). Following CXCL1 stimulation, control neutrophils showed increased fibrinogen binding compared with unstimulated neutrophils (Figure 6G). Neutrophils lacking Csk exhibited even higher fibrinogen binding compared with stimulated littermate control neutrophils. Taken together, these data underscore the participation of Csk in the activity regulation of the 2 key integrins CD11a and CD11b.

### Csk regulates the activity of SFKs through a cAMP-dependent pathway.

**Figure 7:**
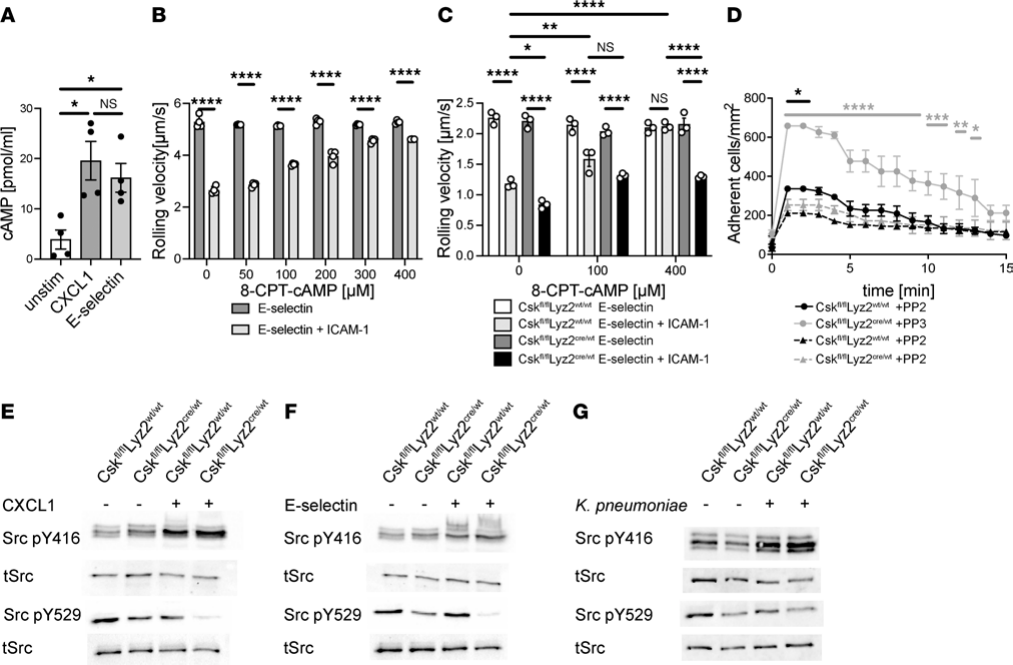
Csk regulates the activity of Src kinases through a cAMP-dependent pathway. (**A**) cAMP levels in cell lysates of murine bone marrow–derived WT neutrophils after stimulation with CXCL1 (2 minutes) or E-selectin (5 minutes). (**B** and **C**) Blood-perfused flow chambers coated with E-selectin or E-selectin/ICAM-1 were used to analyze rolling velocities. Rolling velocity of human neutrophils isolated from whole blood (**B**) and neutrophils isolated from *Csk^fl/fl^Lyz2^wt/wt^* and *Csk^fl/fl^Lyz2^cre/wt^* mice (**C**) after incubation with different concentrations of 8-CPT-cAMP, a cAMP analog and selective activator of the cAMP-dependent PKA. (**D**) Chemokine-induced arrest of neutrophils in postcapillary venules of *Csk^fl/fl^Lyz2^wt/wt^* and *Csk^fl/fl^Lyz2^cre/wt^* mice before and following CXCL1 injection, 30 minutes after intraarterial injection of the specific Src family kinase inhibitor PP2 or the inactive control PP3. Significant differences within the *Csk^fl/fl^Lyz2^wt/wt^* between PP2 and PP3 are marked in black; significant differences within the *Csk^fl/fl^Lyz2^cre/wt^* between PP2 and PP3 are marked in gray. (**E**–**G**) Bone marrow–derived neutrophils were left untreated or were stimulated with CXCL1 for 1 minute, E-selectin for 5 minutes, or serum-opsonized *K*. *pneumoniae* for 1 minute. Lysates were immunoblotted with an Ab against total Src (tSrc) and p-Src Y416 or Y529. (**E**–**G**) Representative Western blots of total lysates of *Csk^fl/fl^Lyz2^wt/wt^* and *Csk^fl/fl^Lyz2^cre/wt^* neutrophils showing the phosphorylation of Src Y416 and Y529 and total amounts of Src. *n* as indicated; *n* = 3–4 for **D**, mean ± SEM. *P < 0.05; **P < 0.01; ****P < 0.0001 by 1-way ANOVA with Tukey’s multiple-comparison test (**A**) or 2-way ANOVA with Šídák’s multiple-comparison test (**B**–**D**).

Integrin activation depends on appropriate signaling responses following primary ligand binding. To assess the signaling mechanisms involved in Csk-dependent integrin activity regulation, we investigated the cAMP/PKA/Csk signaling pathway. First, we stimulated WT neutrophils with either E-selectin or CXCL1 (Figure 7A). Selectin or GPCR stimulation led to a significant increase in cAMP levels (Figure 7A). Increased cAMP levels are known to enhance PKA activation (72). For the following experiments, 8-CPT-cAMP, a cAMP analog and potent and selective activator of the cAMP-dependent PKA (73, 74), was utilized. PKA is known to phosphorylate Csk, thereby increasing its kinase activity (46). To investigate the role of cAMP in integrin activation and slow neutrophil rolling, neutrophils were incubated with different concentrations of 8-CPT-cAMP (Figure 7, B and C). In human neutrophils, rolling on E-selectin and ICAM-1 led to a decrease in rolling velocity compared with E-selectin alone (Figure 7B). This effect was dose-dependently reversed by preincubating the neutrophils with 8-CPT-cAMP (Figure 7B). In murine WT neutrophils, a complete change in the effect was observed (Figure 7C). These findings suggest that the incubation of neutrophils with 8-CPT-cAMP increased the activity of cAMP-dependent PKA activity and thereby boosted Csk activity, resulting in reduced SFK activation. Indeed, *Csk^fl/fl^Lyz2^cre/wt^* neutrophils did not exhibit a full remission of E-selectin–dependent slow rolling when preincubated with 8-CPT-cAMP (Figure 7C). To investigate whether the observed findings were dependent on the modulation of SFK activity by Csk, we conducted chemokine-induced arrest assays (Figure 7D). A body weight–adapted dose of PP2, an SFK-specific inhibitor, or PP3, negative control for PP2, was administered intraarterially 30 minutes prior to chemokine injection. *Csk^fl/fl^Lyz2^cre/wt^* mice exhibited an increased number of adherent cells compared with the control group after CXCL1 injection (Figure 4F). Blocking SFK activity via PP2 dramatically reduced the adhesion of control as well as *Csk^fl/fl^Lyz2^cre/wt^* neutrophils following CXCL1 injection (Figure 7D). The negative control PP3 had no effect on CXCL1-induced adhesion of control as well as *Csk^fl/fl^Lyz2^cre/wt^* neutrophils. Prior to the chemokine application, all groups showed similar baseline values (Figure 7D). These results indicate that the observed phenotype of *Csk*-deficient neutrophils is mediated via regulation of SFK activity. To gain further insight into the underlying mechanisms, we performed biochemical analyses (Figure 7, E–G, and Supplemental Figure 8, A–F). Stimulation of control neutrophils with CXCL1 for 1 minute and E-selectin for 5 minutes induced significant phosphorylation of SFKs at Y416 (Figure 7, E and F, and Supplemental Figure 8, A and B), indicating an enhanced SFK activity (42). In *Csk*-deficient neutrophils, a slight increase in the phosphorylation of SFKs at Y416 was observed at baseline, with a further increase after stimulation with CXCL1 or E-selectin (Figure 7, E and F, and Supplemental Figure 8, A and B). In contrast with stimulation with sterile stimuli, no difference was observed in the phosphorylation of Y416 between *Csk^fl/fl^Lyz2^cre/wt^* and control neutrophils after stimulation with *K*. *pneumoniae* (Figure 7G and Supplemental Figure 8C). After CXCL1 and E-selectin stimulation, *Csk*-deficient neutrophils exhibited dephosphorylation of Y529 (Figure 7, E and F, and Supplemental Figure 8, D and E). *K*. *pneumoniae* stimulation of *Csk*-deficient or control neutrophils did not change the phosphorylation of Y529 (Supplemental Figure 8F). The total protein levels of SFKs did not differ between control and *Csk*-deficient neutrophils.

In summary, we convincingly demonstrated that Csk is a crucial regulator for neutrophil recruitment in vivo. Mechanistically, we propose that selectin- and chemokine-induced increased cAMP levels enhance PKA activity, which in turn activates Csk. Csk itself suppresses the activation of SFKs by phosphorylation of the inhibitory Y529 and inhibition of the phosphorylation of the activating Y416 (Supplemental Figure 8, A, B, D, and E), thereby altering Itgb2 activity (Supplemental Figure 9).

## Discussion

Tight control of neutrophil activation and recruitment is essential to ensure an effective immune response and avoid overshooting reactions. In this study, we demonstrate the pivotal role of the non–receptor tyrosine kinase Csk in the regulation of integrin activation and neutrophil recruitment by modulating the activation status of SFKs.

During bacterial pneumonia, *Csk^fl/fl^Lyz2^cre/wt^* mice exhibited a significant reduction in neutrophil infiltration into lungs. All experiments were conducted using *Lyz2*-driven Cre expression. Although the *Lyz2* promoter can influence expression in cell types beyond neutrophils (61, 75, 76), we implemented multiple controls to ensure that the recruitment defect was specific to neutrophils. To directly link the observed phenotype to neutrophils, we performed lung infection with neutropenic mice after reconstitution with *Csk*-deficient neutrophils and in vitro assays using isolated neutrophils. Furthermore, given that cytokine and chemokine gradients have a substantial effect on immune cell recruitment (77, 78), we measured soluble mediator levels and found no significant difference between *Csk*-deficient mice and their littermate controls. Importantly, the observed neutrophil-specific phenotype was consistent across infections with both Gram-negative and Gram-positive bacteria. Outcomes such as resolution or mortality were not addressed in this study.

*Csk*-deficient mice suffered from higher bacterial burden, which can either result from impaired neutrophil effector functions or the lacking ability of neutrophils to infiltrate the tissue. Assessment of neutrophil effector functions in *Csk*-deficient mice revealed that antibacterial defense mechanisms are not impacted by the absence of Csk, as we observed neither involvement of Csk in the phagocytosis of bacteria, the release of ROS, nor the formation of NETs. Corroborating our observations, previous work demonstrated that knockout of the SFKs Hck, Fgr, and Lyn influences complement-mediated phagocytosis in macrophages (79). Fitzer-Attas et al. reported that the absence of these SFKs in macrophages impaired and in particular delayed Fc-mediated phagocytosis of antibody-coated erythrocytes (79). Therefore, the knockout of Csk may imply that SFK function may be enhanced in Fc-mediated phagocytosis. However, we could not confirm significant differences in the phagocytosis of antibody-opsonized bacteria in neutrophils. Regarding NET formation, a previous study has demonstrated that incubation of human neutrophils with PP2, which leads to inhibition of SFKs, does not affect NET release after PMA stimulation, but inhibits β-glucan–induced NET formation (80). In our experimental set up, NET release following stimulation with PMA or with *K*. *pneumoniae* was comparable in *Csk*-deficient and control neutrophils. Thus, the observations during bacterial pneumonia are not due to an impaired ability of *Csk*-deficient neutrophils to combat invading bacteria but rather result from deficient neutrophil recruitment in the absence of Csk.

We found that *Csk*-deficient neutrophils encounter difficulties in successful infiltration of the lung tissue. As we have demonstrated in several experiments, adhesion of neutrophils is significantly increased in *Csk*-deficient mice. Accordingly, Sperandio et al. demonstrated that knockout of the SFKs Hck, Fgr, and Lyn reduces adhesion of neutrophils in the venules of the TNF-stimulated cremaster muscle compared with WT mice (34), demonstrating the crucial contribution of SFKs to adhesion strengthening. Postadhesion strengthening mediates stable interactions between neutrophils and the vessel wall, allowing neutrophils to withstand the shear forces of the blood flow and subsequent neutrophil migration (81–84). Indeed, systemic administration of PP2, an SFK-specific inhibitor, leads to a reduced adhesion of neutrophils in the venules of the murine cremaster muscle after CXCL1 injection. This highlights that effects observed in our model are concordantly mediated by direct regulation of SFKs by Csk. It is well described that the function of SFKs exhibit redundancy. The elimination of multiple kinases is required to disrupt downstream signaling (85, 86). The knockout of Csk as a crucial part of the upstream signaling pathway may thus result in the alteration of all relevant downstream SFKs.

Adequate recruitment of neutrophils relies on precise control of integrin functionality, specifically CD11a and CD11b that are required for the adhesion of neutrophils to the endothelium (21). Our FACS analysis and murine flow chamber results provide compelling evidence that neutrophilic Csk negatively regulates the activation of CD11a and CD11b. Overactivation of CD11a and CD11b can exacerbate tissue damage and contribute to sclerosis, inflammatory conditions, or autoimmune diseases (80, 87–91). In concordance with our results, Thomas et al. previously showed in an in vitro system that the activation of CD11b in *Csk*-deficient granulocytes is fully suppressed by the administration of PP2 (38). We can specify these findings to neutrophils, verify them in multiple in vivo models, and extend them to the human system by using HL-60 cells. During bacterial pneumonia, *Csk*-deficient neutrophils exhibit increased adhesion, but are unable to effectively transmigrate across the endothelium to reach the infected lung tissue; instead, they remain in the intravascular space. We have verified that these functional differences are not due to modulated receptor expression or baseline differences between the 2 compared groups. Integrin activation can be induced by selectin- and chemokine-mediated signaling (92). While Thomas et al. investigated the effect of Csk depletion in PMA- and fMLP-mediated integrin functions, we also analyzed the role of Csk in selectin-induced pathways (38). Selectins are critical for the initiation of neutrophil recruitment by mediating rolling along the vessel wall (6). Beyond that, selectin engagement triggers intracellular signaling pathways leading to the activation of Itgb2 (6, 93, 94). Using various murine and human flow chamber systems, we show that Csk negatively regulates selectin-mediated rolling velocities and integrin activation by modulation of SFKs.

CXCL1 acts via GPCR and E-selectin via PSGL1 (6, 95). Mechanistically, we demonstrate that neutrophil stimulation with CXCL1 or E-selectin elevates cAMP levels in WT neutrophils and thus activates distinct signaling pathways. This increase in cAMP leads to a corresponding neutrophil response, as evidenced by higher rolling velocities in various flow chamber setups. Notably, this response is completely absent in *Csk*-deficient mice, demonstrating that cAMP levels regulate Csk and integrin activity. Additionally, we show here that this activation depends on cAMP levels. In further studies using PP2, we confirm that the effects of Csk knockout results from overactivation of SFKs. We demonstrate that SFK phosphorylation sites in neutrophils are modulated by Csk. Csk not only regulates SFK phosphorylation at Y529 but also significantly affects Y416 phosphorylation in neutrophils, thereby modulating pivotal neutrophil behavior. Ongoing research will explore how Csk influences Y416 phosphorylation through active or passive signaling pathways, as its effects beyond Y529 are not yet fully understood.

In recent years, researchers have made progress in the development of various Csk inhibitors. Modulation of Csk has emerged as a potential therapeutic target for a variety of immune cells and diseases. Oncogenic overexpression of Csk is known to be important in breast cancer progression and a study in 2024 evaluated several Csk inhibitors, one of which inhibited growth in 3 different human breast cancer cell lines (96). Lu et al. showed that chemical-genetic inhibition of Csk in mouse B cells suppresses B cell receptor signaling, which is important for maintaining peripheral tolerance (97). Additionally, oral administration of a Csk inhibitor to mice resulted in T cell activation in the spleen within 6 hours after administration (98). In conclusion, Csk modulation appears to be an interesting target in several diseases, including cancer.

In summary, our study demonstrates the pivotal role of Csk in E-selectin– and CXCL1-mediated activation of the 2 key integrins, CD11a and CD11b, representing key elements during the different steps of the neutrophil recruitment cascade, especially neutrophil adhesion. In brief, Csk is required to regulate neutrophil responses during pulmonary infections. Further investigations are required to ascertain whether Csk could serve as a potential target for chemical modulation of neutrophil recruitment during inflammatory conditions such as bacterial pneumonia.

## Methods

### Sex as a biological variable.

Our study examined male and female mice and blood donors, and similar findings are reported for both sexes.

### Mice.

*Csk^fl/fl^Lyz2^wt/wt^*, *Csk^fl/fl^Lyz2^cre/wt^*, *Mcl-1^fl/fl^Ly6G^cre/wt^*, and *Mcl-1^fl/fl^Ly6G^cre/cre^* mice were used throughout this study (99–101). Animals were maintained in a specific pathogen–free facility at the University of Muenster.

### Lung infection with K. pneumoniae.

Overnight cultures of *K*. *pneumoniae* (ATCC strain 13883) were grown in Tryptic Soy medium. Mice were anesthetized by intraperitoneal injection of ketamine (125 mg/kg; WDT, bela-pharm) and xylazine (12.5 mg/kg; Elanco). The trachea was exposed and a 50 μL inoculum (4 × 10^7^ bacteria in saline) was administered via a 27-gauge needle. Sham-operated mice received the same surgical procedure and 50 μL inoculum of sterile solution administered intratracheally. After 24 hours or 36 hours, mice were sacrificed, and lungs were lavaged 4 times with 0.7 mL saline solution. CFUs in the lungs, BALF, blood, and spleen were counted by serial plating on Tryptic Soy agar plates (102). Cells in the lungs and BALF were analyzed by flow cytometry (FACSCanto II, BD Biosciences). Neutrophils were gated as CD45^+^CD11b^+^CX3CR1^–^Ly6G^+^Gr-1^+^ using antibodies against CD45 (clone 30-F11, BD Biosciences), CD11b (clone M1/70, BD Biosciences), CX3CR1 (clone SA011F11, BioLegend), Ly6G (clone 1A8, BioLegend), and Gr-1 (clone RB6-8C5, purified from hybridoma supernatant; ref. 103). Monocytes were gated as CD45^+^CD11b^+^CX3CR1^+^Ly6C^hi^Ly6G^–^Gr-1^–^ using the antibodies above as well as an antibody against Ly6C (clone HK1.4, BioLegend). Alveolar macrophages were gated as CD45^+^CD64^+^F4/80^+^MARCO^+^SiglecF^hi^ using antibodies against CD45 (clone 30-F11, BioLegend), CD64 (clone X54-5/7.1, BioLegend), F4/80 (clone BM8, BioLegend), MARCO (clone 579511, R&D Systems), and SiglecF (clone S17007L, BioLegend). Natural killer cells were gated as CD45^+^CD27^+^CD335^+^ using antibodies against CD45 (clone 30-F11, BD Bioscience), CD27 (clone LG.3A10, BioLegend), and CD335 (clone 29A1.4, BioLegend). Dendritic cells were gated as CD45^+^CD27^–^CD24^+^CD11c^+^MHCII^+^ using antibodies against CD45 (clone 30-F11, BD Bioscience), CD27 (clone LG.3A10, BioLegend), CD24 (clone M1/69, BioLegend), CD11c (clone HL3, BD Biosciences), and MHCII (clone M5/114.15.2, BioLegend). DAPI (D9542, Sigma-Aldrich) and eFluor 780 (Invitrogen) were used as viability dyes. Lung, BALF, and serum samples were analyzed with LEGENDplex Mouse Inflammation Panel 13-plex (BioLegend). For reconstitution experiments, the same procedure was performed with *Mcl-1^fl/fl^Ly6G^cre/wt^* mice receiving neutrophils, freshly isolated with a 2-layer gradient, injected intravenously briefly prior to intratracheal injection.

### Lung infection with S.

*aureus*. *S*. *aureus* strain AH1263 (USA CA-MRSA ErmS) was cultivated on Columbia 5% blood agar plates (Thermo Fisher Scientific) and grown in brain heart glucose bouillon (BHI, Roth) (60, 104). CFUs were determined as recently published (60). The surgical procedure was performed as described above. Mice were infected with 2 × 10^8^ viable *S*. *aureus* per mouse (60). After 24 hours, the mice were sacrificed and the same experimental processing as described above was carried out.

### Phagocytosis.

Live *K*. *pneumoniae* bacteria were labeled using a pHrodo Red Phagocytosis Particle Labeling Kit for Flow Cytometry (Thermo Fisher Scientific) following the manufacturer’s protocol. Labeled bacteria were opsonized with mouse serum at a 1:1 ratio for 30 minutes at 37°C or with IgG (normal mouse IgG, sc-2025, Santa Cruz Biotechnology) at a 1:2 ratio overnight at 4°C on a rolling wheel (105). Neutrophils were incubated with opsonized bacteria at 37°C for 1 or 2 hours at a ratio of 1:10. Negative controls were kept on ice. Where indicated, PP2, a specific inhibitor of SFKs (ab120308, Abcam), or PP3, a negative control for PP2 (ab120617, Abcam), was used a dose of 10 μM (25). Neutrophils were stained with Alexa Fluor 488–conjugated anti-Ly6G (clone 1A8, BioLegend) and flow cytometry (FACSCanto II, BD Biosciences) was performed.

### Oxidative burst.

Isolated neutrophils were applied to fibrinogen-precoated (from bovine plasma, 150 μg/mL, 3 hours, RT; Sigma-Aldrich) 96-well plates (Immunolon-4 HBX, Thermo Fisher Scientific) with 1 mM CaCl_2_, 1 mM MgCl_2_, 0.1 mM cytochrome *c* (Sigma-Aldrich), and 50 ng/mL TNF (BioLegend) or *K*. *pneumoniae* bacteria. Overnight cultures of *K*. *pneumoniae* were collected at an OD of 0.9 and prepared at an MOI of 50 and opsonized with mouse serum at a 1:1 ratio for 30 minutes at 37°C. Absorbance at 490 and 550 nm was recorded every 2 minutes for 90 minutes at 37°C in a plate reader. For calculations, each wavelength value was corrected by its superoxide dismutase control value (102).

### Measurement of NET formation by flow cytometry.

Neutrophils were stimulated with 100 nM PMA (Sigma-Aldrich) or *K*. *pneumoniae* (OD 0.9, MOI 50) for 1 or 3 hours (37°C, 5% CO_2_) and stained afterwards with anti-MPO (1 μg/mL; clone 2D4, Abcam), anti-Ly6G (clone 1A8, BioLegend), and 0.5 μM Sytox Dead Cell stain (S34857, Invitrogen). Fluorescence intensity was measured by flow cytometry (FACSCanto II, BD Biosciences).

### Intravital microscopy.

The cremaster muscle was prepared for intravital imaging as previously described (106). Postcapillary venules with a diameter between 20 and 40 μm were investigated. To determine neutrophil adhesion, 500 ng CXCL1 (PeproTech) was injected via the carotid artery. The number of adherent cells prior and following CXCL1 injection was analyzed. In other experiments, the SFK-specific inhibitor PP2 (10 μg/kg BW; ab120308, Abcam) or the inactive control PP3 (10 μg/kg BW; ab120617, Abcam) was injected intraarterially 30 minutes prior to recording. To determine selectin-mediated slow rolling, adhesion, and transmigration in vivo, mice were injected intrascrotally with 500 ng TNF (BioLegend) 2 hours before the preparation of the cremaster muscle. Intravital microscopy was performed on an upright microscope (Axioskop, Zeiss) with a 40×/0.75 NA saline immersion objective. Neutrophil rolling velocity and adhesion were determined by transillumination intravital microscopy, whereas extravasation was investigated by reflected light oblique transillumination (RLOT) microscopy using a 20× objective, as previously described (107). Recorded images were analyzed using ImageJ (NIH) and SlideBook 6 Reader (Intelligent Imaging). Emigrated cells were determined in an area 75 × 100 μm to each side of a vessel (representing 1.5 × 10^4^ μm^2^ tissue area). The microcirculation was recorded using a digital camera (Sensicam QE). Blood flow centerline velocity was measured using a dual photodiode sensor system (Circusoft Instrumentation). Centerline velocities were converted to mean blood flow velocities, as previously described (24, 108).

### Murine flow chamber systems.

To investigate the rolling velocity of neutrophils, we used a previously published flow chamber system (109, 110). Rectangular glass capillaries (20 × 200 μm) were coated either with murine E-selectin (2.5 μg/mL; 575-ES-100, R&D Systems) or P-selectin (20 μg/mL; 737-PS-200, R&D Systems) alone or in combination with ICAM-1 (2 μg/mL in combination with E-selectin, 5 μg/mL in combination with P-selectin; R&D Systems) for 2 hours and then blocked for 2 hours using 1% casein (Thermo Fisher Scientific). One side of the chamber was connected to PE 10 tubing (Becton Dickinson) and inserted into the murine carotid artery. The other side of the chamber was connected to PE 50 (Becton Dickinson) tubing and used to control the wall shear stress in the capillary. To investigate chemokine-induced adhesion in vitro (111), glass capillaries were coated with murine P-selectin (50 μg/mL; R&D Systems) and ICAM-1 (15 μg/mL; R&D Systems) or P-selectin and ICAM-1 in combination with CXCL1 (25 μg/mL; PeproTech). After 5 minutes of constant blood flow, representative fields of view were recorded for 1 minute using an SW40/0.75 NA objective and a digital camera (Sensicam QE) to quantify adhesion.

### Intravascular crawling assay.

By using intravital microscopy, the intravascular crawling behavior was determined as described previously (26). Anti–Gr-1 antibody (750 ng; clone RB6-8C5, purified from hybridoma supernatant; ref. 103), labeled with Alexa Fluor 488 (Thermo Fisher Scientific), was injected intraarterially directly prior the experiment. Following preparation and exteriorization, the cremaster muscle was superfused with CXCL2 (5 nM; R&D Systems) and time-lapse microscopy was performed.

### In vitro chemotaxis assay.

In vitro chemotaxis was analyzed as described previously (108). Neutrophils were seeded on fibronectin-coated (from bovine plasma, 50 μg/mL, 37°C, overnight; Sigma-Aldrich) chemotaxis μ-slides (Ibidi). A CXCL1 gradient was applied by diffusion of a Patent Blue–colored (1:100; Sigma-Aldrich) CXCL1 solution (10 μg/mL; PeproTech) in one reservoir of the slide according to the manufacturer’s instructions. Cell movement was recorded on a microscope platform (37°C, 5% CO_2_, BioTek Lionheart) over a period of 90 minutes by using time-lapse microscopy (3 frames/min). Cells were analyzed with Manual Tracking (ImageJ, NIH) and the Chemotaxis plug-in (Ibidi) (108).

### Reporter antibody adhesion flow chambers.

Adhesion flow chamber experiments were carried out as reported previously (25). Protein G–coated (500 μg/mL; EMD) glass capillaries were coated with human E-selectin (6.6 μg/mL; ADP1-050, R&D Systems) and IgG1 (25 μg/mL; BD Pharmingen) or KIM127 antibody (25 μg/mL; purified from hybridoma; ref. 67) for 1 hour and blocked with 1% casein (Thermo Fisher Scientific). In other experiments, capillaries were coated with human P-selectin (20 μg/mL; 137-PS-050, R&D Systems), IL-8 (50 μg/mL; PeproTech), and IgG1 (5 μg/mL; Santa Cruz Biotechnology) or mAb24 (5 μg/mL; refs. 112,113; gift from Nancy Hogg, Cancer Research UK London Research Institute) or CBRM1/5 antibodies (40 μg/mL; 301402, BioLegend). HL-60 cells (Sigma-Aldrich) were resuspended in human plasma. The flow chamber was perfused with the cell suspension for 2 minutes and washed with PBS for 1 minute. In representative images, the number of cells per field of view was determined.

### Soluble ICAM-1 and fibrinogen binding assays.

ICAM-1 and fibrinogen binding assays were performed as described in an earlier publication (24). Neutrophils were isolated using a 2-layer gradient (114) and incubated overnight at 37°C in 5% CO_2_ to recover from the additional washing steps that are required to achieve high purity but may induce cellular stress. To assess CD11a-specific ICAM-1 binding, neutrophils were preincubated with a functional blocking anti-CD11b antibody (clone M1/70; 10 μg/mL) and stimulated with CXCL1 (100 ng/mL, 3 minutes, 37°C; PeproTech) in the presence of ICAM-1/Fc (20 μg/mL; R&D Systems) and an APC-conjugated anti-human IgG1 antibody (Fc-specific, 9042-11, Southern Biotechnology). Neutrophils were stained with FITC-conjugated anti-Ly6B.2 antibody (7/4, MCA771FB, Bio-Rad). CD11a-specific binding to ICAM-1/Fc was measured by flow cytometry (FACSCanto II, BD Biosciences). To investigate CD11b’s affinity for fibrinogen, neutrophils were incubated with Alexa Fluor 647–conjugated fibrinogen (150 μg/mL, 10 minutes, 37°C; Thermo Fisher Scientific) and stimulated with CXCL1 (100 ng/mL, 10 minutes, 37°C; PeproTech). Neutrophils were stained with FITC-conjugated anti-Ly6B.2 antibody (7/4, MCA771FB, Bio-Rad). Fluorescence intensity was measured by flow cytometry (FACSCanto II, BD Biosciences).

### Human and murine flow chamber assay after 8-CPT-cAMP stimulation.

Flow chamber assays were performed as described previously (25). Glass capillaries were coated with E-selectin (3.5 μg/mL; 575-ES-100 and ADP1-050, R&D Systems) or E-selectin/ICAM-1 (3.5/3.5 μg/mL) for 2 hours. Chambers were blocked with 1% casein (Thermo Fisher Scientific) for 1 hour. Human or murine whole blood from *Csk*-deficient or control mice was isolated as described above. Cells were incubated with different concentrations of 8-CPT-cAMP (Abcam) for 20 minutes and resuspended in plasma. The flow chamber was perfused with cell suspension for 2 minutes at a constant shear stress of 5–6 dynes/cm^2^ and washed with PBS for 1 minute. One representative field of view was recorded for 1 minute using an SW40/0.75 NA objective and a digital camera (Sensicam QE) to determine the rolling velocities of the cells.

### Western blot analysis.

Neutrophils were incubated under rotating conditions (65 rpm) for 5 minutes on uncoated or E-selectin–coated (3 μg/mL; 575-ES-100, R&D Systems) coverslips in plates or stimulated with CXCL1 (100 ng/mL, 37°C; PeproTech) for 1 minute. *K*. *pneumoniae* were prepared and opsonized with mouse serum as described above and stimulation lasted 1 minute. Cells were lysed in RIPA buffer and boiled with Laemmli sample buffer (10 minutes, 95°C). Cell lysates were run in 10% SDS-PAGE and immunoblotted using antibodies against tSrc (L4A1, Cell Signaling Technology), p-SFK Y416 (D49G4, Cell Signaling Technology), and p-SKF Y529 (44-662G, Invitrogen). Immunoblots were developed using an ECL system (Cytiva). Densitometric quantification was performed using Image Lab software (Bio-Rad).

### Statistics.

The number of experimental repeats is specified in the corresponding figure legend and statistical analysis was based on single experiments. Statistical analysis was performed with GraphPad Prism 9. To test normal distribution, the Shapiro-Wilk test was used. Differences between the groups were evaluated by 1-way or 2-way analysis of variance (ANOVA), Student-Newman-Keuls test, rank-sum test, or *t* test as appropriate. Data are presented as mean ± SEM, and *P* values of less than 0.05 were considered statistically significant.

### Study approval.

The Animal Care and Use Committee of the University of Muenster and the institutional review board of North Rhine-Westphalia (Germany) approved animal experiments (AZ84-02.04.2016.A438, AZ81-02.04.2023.A065, A20.014, T23.055). Ethical approval for human blood samples was granted by the Ethics Committee of the Medical Association Westfalen-Lippe (AZ 2012-021-f-S). Written informed consent was received prior to participation.

### Data availability.

All data values of the figures are provided in the Supporting Data Values file. For original data, please contact the corresponding author.

## Author contributions

WA, LS, JNH, MO, KT, HB, AM, and AC performed experiments and analyzed and interpreted data. PL and BB performed experiments. WA and AC wrote the manuscript. AM, OS, and AZ contributed to writing the manuscript. AC and AZ conceived the study. AZ supervised the study.

## Supplementary Material

Supplemental data

Unedited blot and gel images

Supporting data values

## Version 1 06/10/2025

In-Press Preview

## Version 2 07/22/2025

Electronic publication
