# Supplementary material for: Csk-mediated Src family kinase regulation dampens neutrophil infiltration during pulmonary infection
Source: JCI Insight. 2025 Jun 10;10(14):e188323. doi: 10.1172/jci.insight.188323 (PMC12288981; doi:10.1172/jci.insight.188323)
Supplement: Supplemental data [file jciinsight-10-188323-s029.pdf]

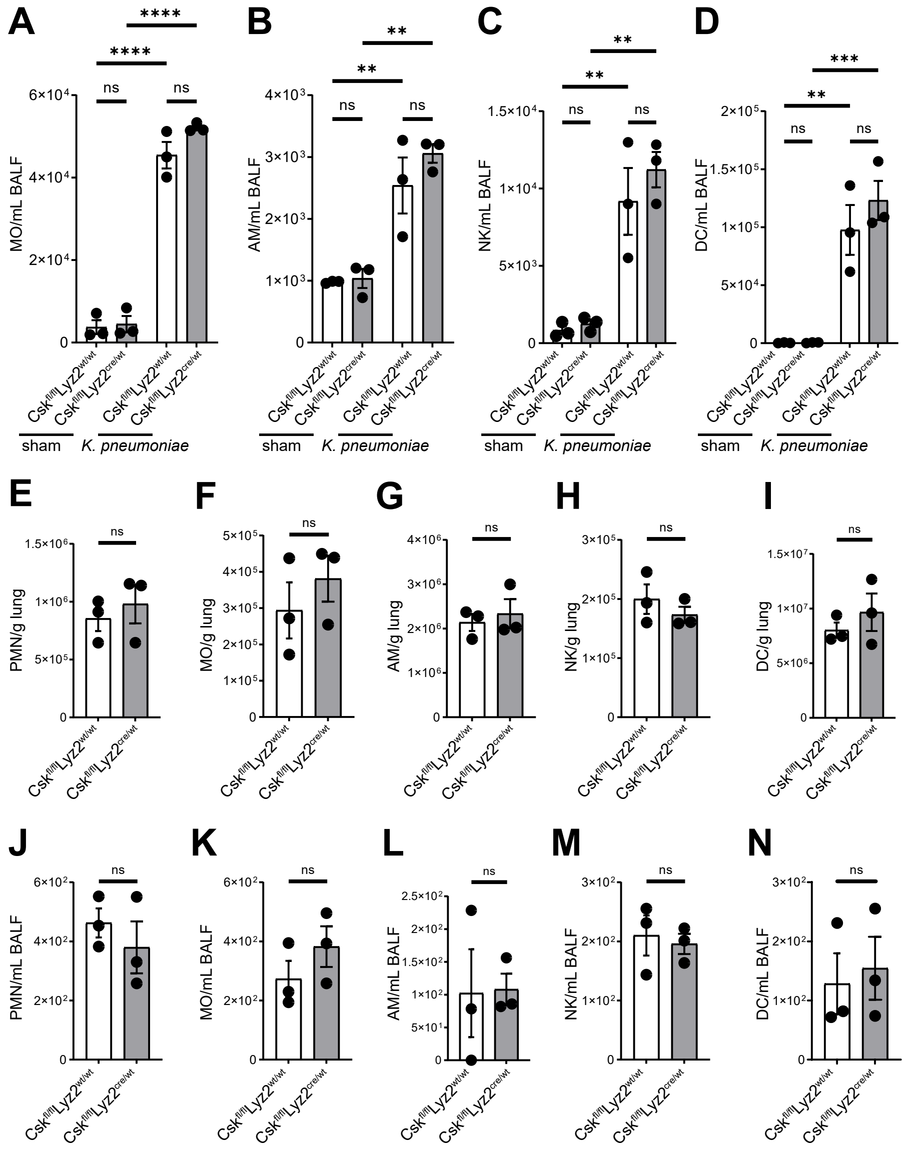

Supplemental Figure 1: Immune cell populations in the BALF and lungs of *Csk^fl/fl Lyz2^wt/wt* and *Csk^fl/fl Lyz2^cre/wt* mice after 24 h after *K. pneumoniae* infection and under steady-state conditions. (**A**-**D**) Monocytes (MO; $CD45^{+}CD11b^{+}CX3CR1^{+}Ly6C^{hi}Ly6G^{-}Gr\text{-}1^{-}$) (A), alveolar macrophages (AM; $CD45^{+}CD64^{+}F4/80^{+}MARCO^{+}SiglecF^{hi}$) (B), natural killer cells (NK; $CD45^{+}CD27^{+}CD335^{+}$) (C) and dendritic cells (DC; $CD45^{+}CD27^{-}CD24^{+}CD11c^{+}MHCII^{+}$) (D) in the BALF 24 h after *K. pneumoniae* lung infection. (**E**-**N**) Cell counts in the lung and BALF under steady state conditions including neutrophils (PMN; $CD45^{+}CD11b^{+}CX3CR1^{-}Ly6G^{+}Gr\text{-}1^{+}$), monocytes, alveolar macrophages, natural killer cells and dendritic cells (n as indicated, mean ± SEM, 1-way-ANOVA (A-D) or Student's t test (E-N), *p<0.05; **p<0.01; ***p<0.001; ****p<0.0001).

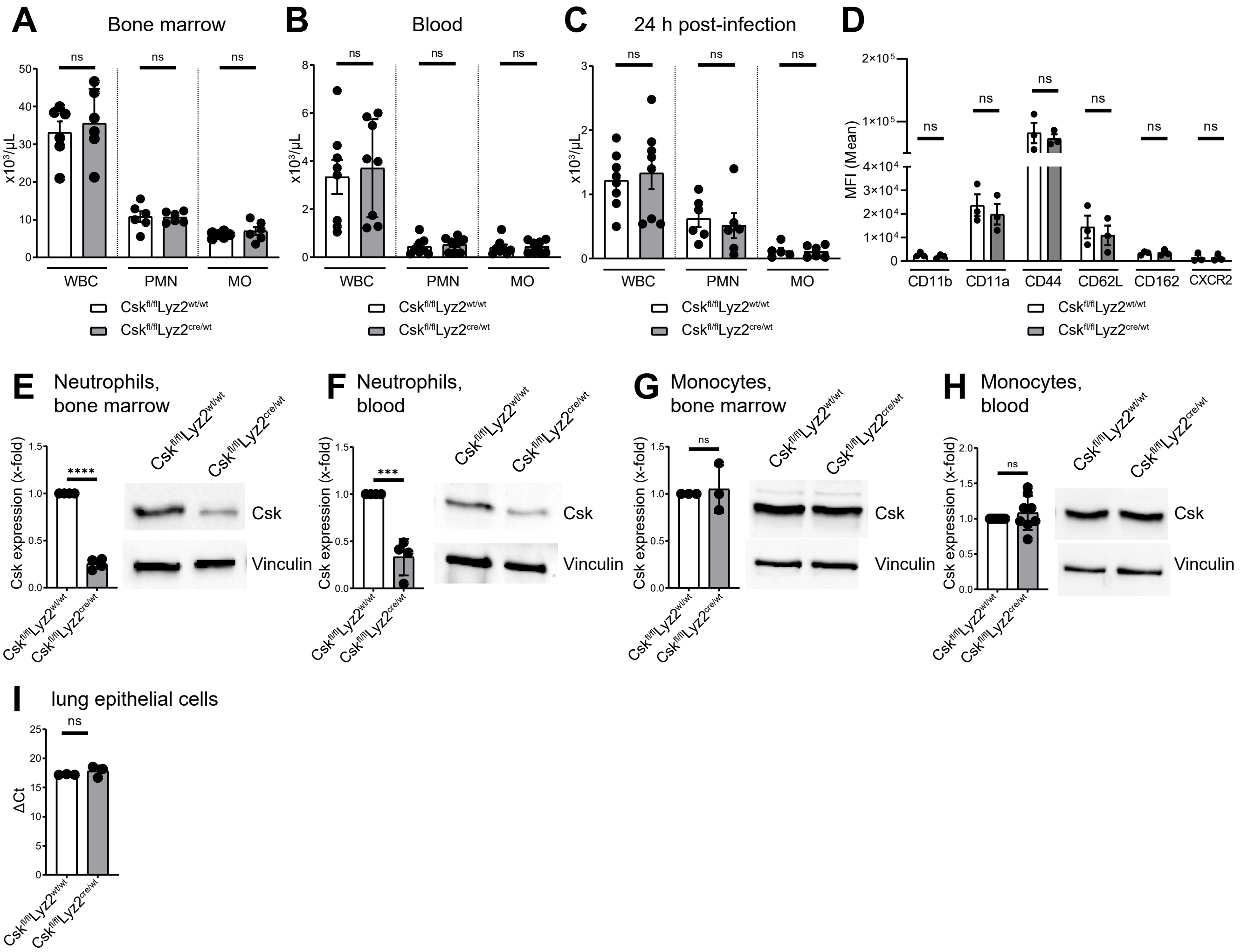

Supplemental Figure 2: Effect of Csk knockout in Lyz2-cre mice. (**A**-**C**) White blood cell (WBC), neutrophil (PMN) and monocyte (MO) counts in the bone marrow (A) and blood under steady state conditions (B) and 24 h after infection with *K. pneumoniae* (C) in *Csk$^{fl/fl}$Lyz2$^{wt/wt}$* and *Csk$^{fl/fl}$Lyz2$^{cre/wt}$* mice, determined via Sysmex analysis. **(D)** Surface expression levels of CD11b, CD11a, CD44, CD62L, CD162, CXCR2 of whole blood neutrophils from *Csk$^{fl/fl}$Lyz2$^{wt/wt}$* and *Csk$^{fl/fl}$Lyz2$^{cre/wt}$* mice. (**E**-**F**) Csk expression in bone marrow derived (E) and circulating (F) neutrophils. (**G**-**H**) Csk expression in bone marrow derived (G) and circulating (H) monocytes, determined by Western Blot Analysis. (**I**) Csk expression in lung epithelial cells, determined by qPCR. (n as indicated, mean ± SEM, Student's t test, *p<0.05; **p<0.01; ***p<0.001; ****p<0.0001).

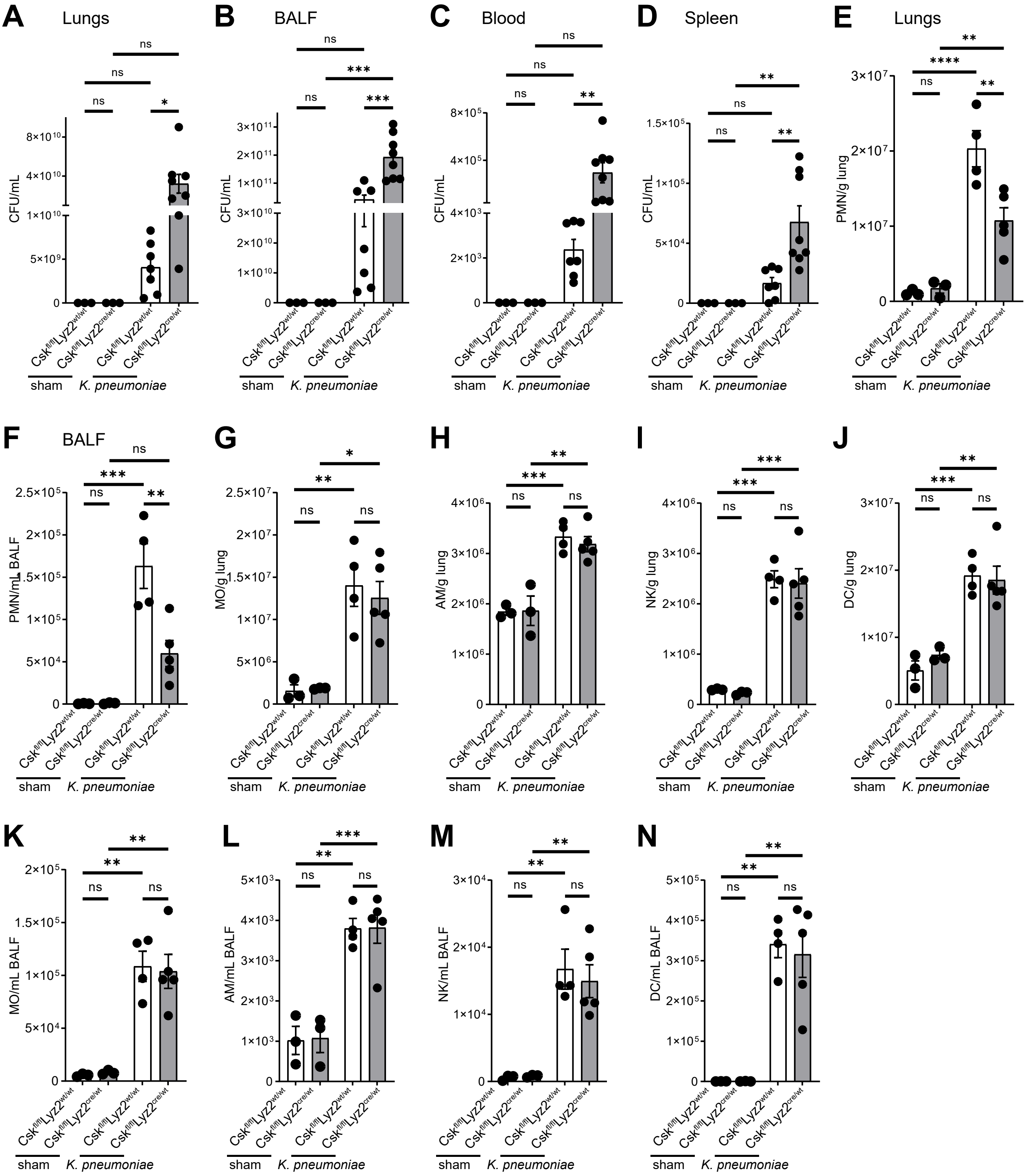

Supplemental Figure 3: Csk is crucial for neutrophil recruitment and bacterial clearance in *K. pneumoniae* induced pneumonia after 36 h. (**A-N**) $Csk^{fl/fl}Lyz2^{wt/wt}$ and $Csk^{fl/fl}Lyz2^{cre/wt}$ mice were subjected to *K. pneumoniae* intratracheal injection or sham surgery. (A-D) Bacterial burden regarding the colony forming units (CFUs) in lungs (A), bronchoalveolar fluid (BALF) (B), blood (C) and spleen (D). (E-F) Neutrophil (PMN; $CD45^+CD11b^+CX3CR1^-Ly6G^+Gr\text{-}1^+$) recruitment into lungs and BAL. (H-N) Immune cell recruitment into lungs and BALF were determined 36 h after *K. pneumoniae* injection. Cell count of monocytes (MO; $CD45^+CD11b^+CX3CR1^+Ly6C^{hi}Ly6G^-Gr\text{-}1^-$), alveolar macrophages (AM; $CD45^+CD64^+F4/80^+MARCO^+SiglecF^{hi}$), natural killer cells (NK; $CD45^+CD27^+CD335^+$) and dendritic cells (DC; $CD45^+CD27^-CD24^+CD11c^+MHCII^+$) (n as indicated, mean ± SEM, 1-way-ANOVA, *p<0.05; **p<0.01; ***p<0.001; ****p<0.0001).

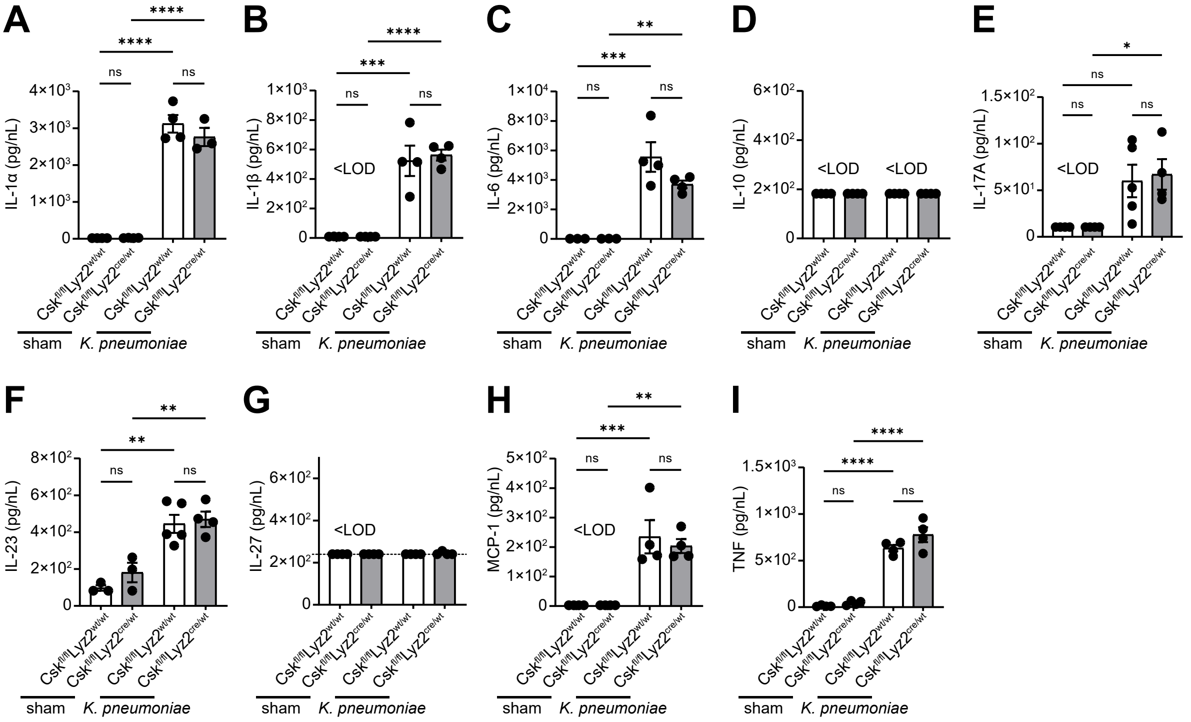

Supplemental Figure 4: LEGENDplex™ analysis of murine cytokines and chemokines in lung samples of sham operated mice and mice after *K. pneumoniae* infection. (**A-I**) Lung samples from *Csk*$^{fl/fl}$*Lyz2*$^{wt/wt}$ and *Csk*$^{fl/fl}$*Lyz2*$^{cre/wt}$ were analyzed 24 h after intratracheal injection of *K. pneumoniae* or sham surgery. Quantification of IL-1alpha (A), IL-1beta (B), IL-6 (C), IL-10 (D), IL-17A (E), IL-23 (F), IL-27 (G), MCP-1 (H) and TNF (I). (n as indicated, mean ± SEM, 1-way-ANOVA, *p<0.05; **p<0.01; ***p<0.001; ****p<0.0001; LOD: limit of detection).

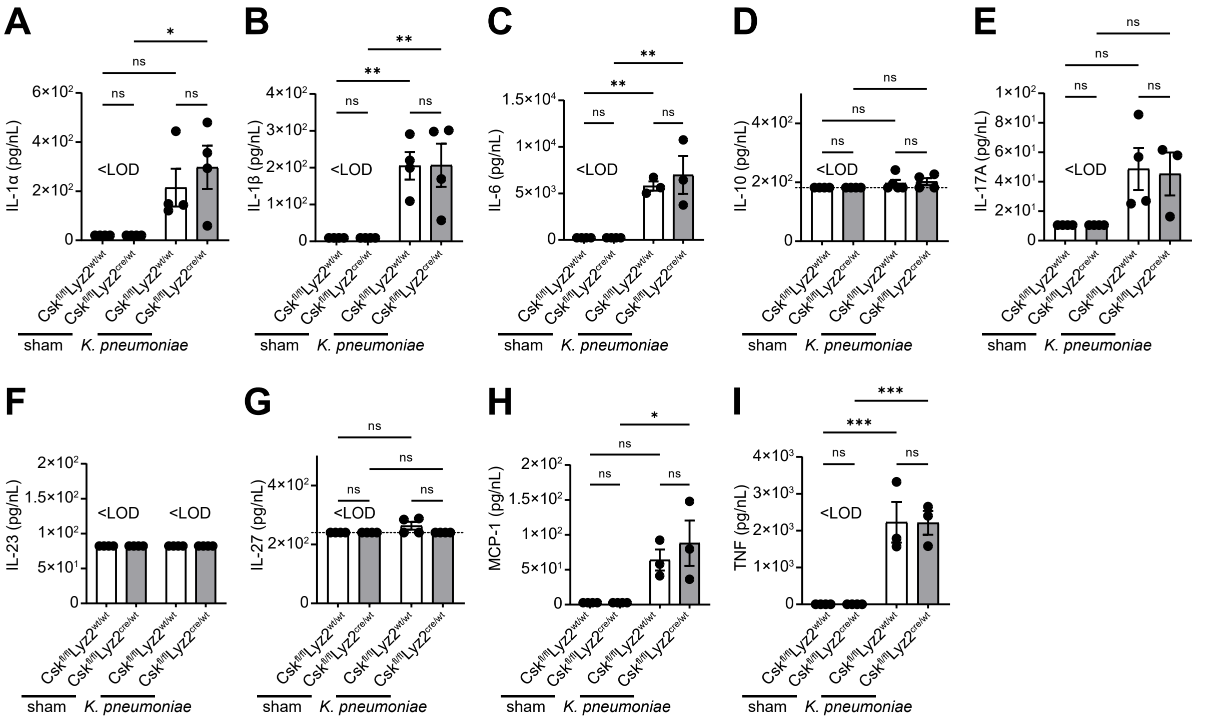

Supplemental Figure 5: LEGENDplex™ analysis of murine cytokines and chemokines in BALF samples of sham operated mice and mice after *K. pneumoniae* infection. (**A-I**) Bronchoalveolar fluid (BALF) samples from *Csk$^{fl/fl}$Lyz2$^{wt/wt}$* and *Csk$^{fl/fl}$Lyz2$^{cre/wt}$* were analyzed 24 h after intratracheal injection of *K. pneumoniae* or sham surgery. Quantification of IL-1alpha (A), IL-1beta (B), IL-6 (C), IL-10 (D), IL-17A (E), IL-23 (F), IL-27 (G), MCP-1 (H) and TNF (I). (n as indicated, mean ± SEM, 1-way-ANOVA, *p<0.05; **p<0.01; ***p<0.001; LOD: limit of detection).

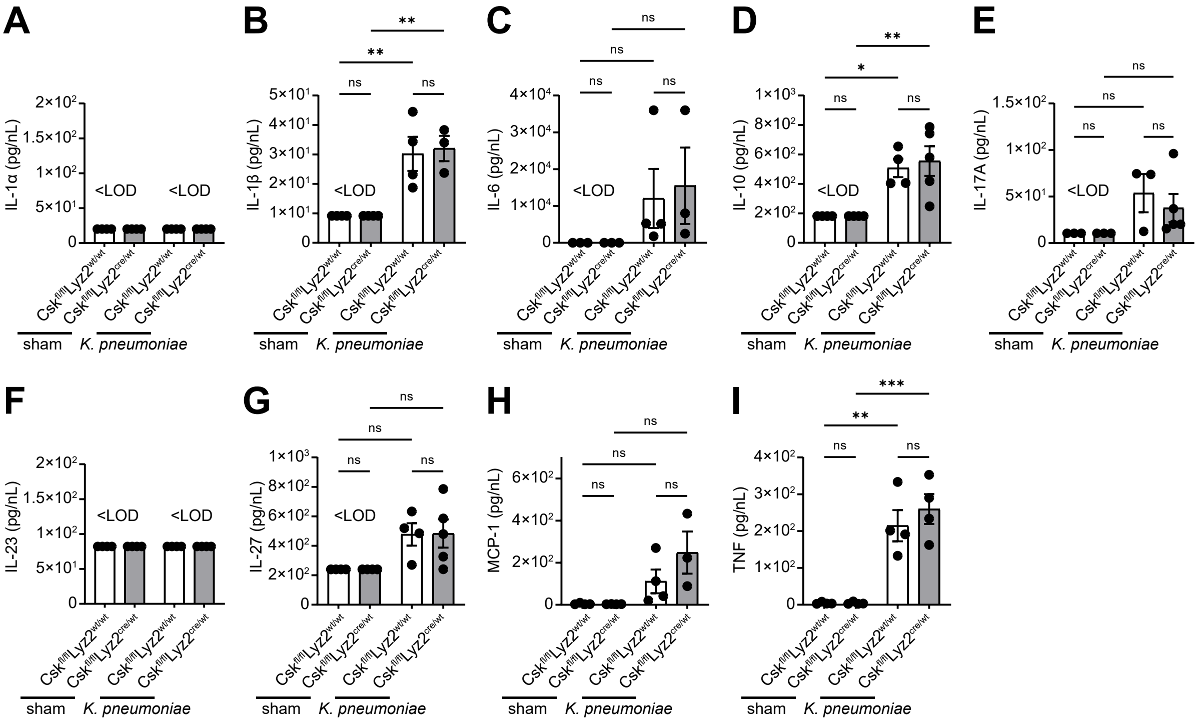

Supplemental Figure 6: LEGENDplex™ analysis of murine cytokines and chemokines in serum samples of sham operated mice and mice after *K. pneumoniae* infection. (**A-I**) Serum samples from *Csk*$^{fl/fl}$*Lyz2*$^{wt/wt}$ and *Csk*$^{fl/fl}$*Lyz2*$^{cre/wt}$ were analyzed 24 h after intratracheal injection of *K. pneumoniae* or sham surgery. Quantification of IL-1alpha (A), IL-1beta (B), IL-6 (C), IL-10 (D), IL-17A (E), IL-23 (F), IL-27 (G), MCP-1 (H) and TNF (I). (n as indicated, mean ± SEM, 1-way-ANOVA, *$p<0.05$; **$p<0.01$; ***$p<0.001$; LOD: limit of detection).

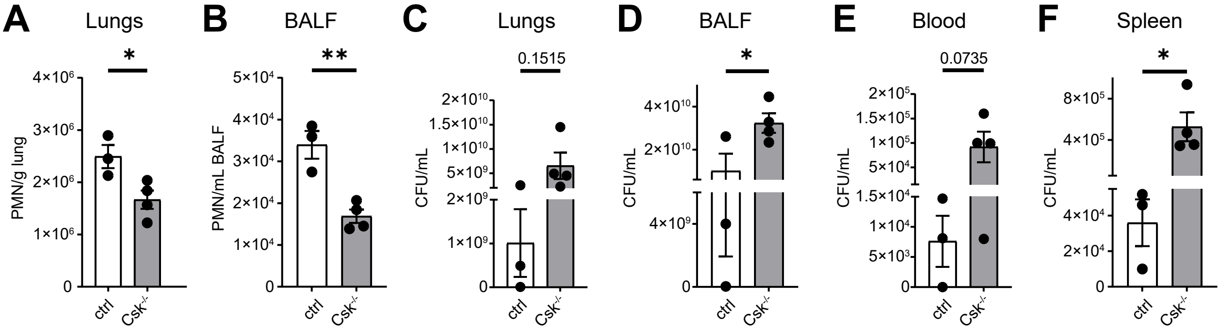

Supplemental Figure 7: Reconstitution of neutropenic *Mcl-1$^{fl/fl}$Ly6G$^{cre/wt}$* mice with *Csk*-deficient neutrophils leads to reduced neutrophil recruitment during *K. pneumoniae* induced pneumonia. (**A**-**F**) *Mcl-1$^{fl/fl}$Ly6G$^{cre/wt}$* mice received fresh isolated control and *Csk*-deficient neutrophils intravenously and were subjected to *K. pneumoniae* intratracheal injection. Neutrophil (PMN; $CD45^{+}CD11b^{+}CX3CR1^{-}Ly6G^{+}Gr\text{-}1^{+}$) recruitment into lungs (A) and BALF (B) were determined 24 h after *K. pneumoniae* injection. Bacterial burden regarding the colony forming units (CFUs) in lungs (C), BALF (D), blood (E) and spleen (F). (n = 3-4 mice per genotype, mean ± SEM, Student's t test, *p<0.05; **p<0.01; ***p<0.001; ****p<0.0001).

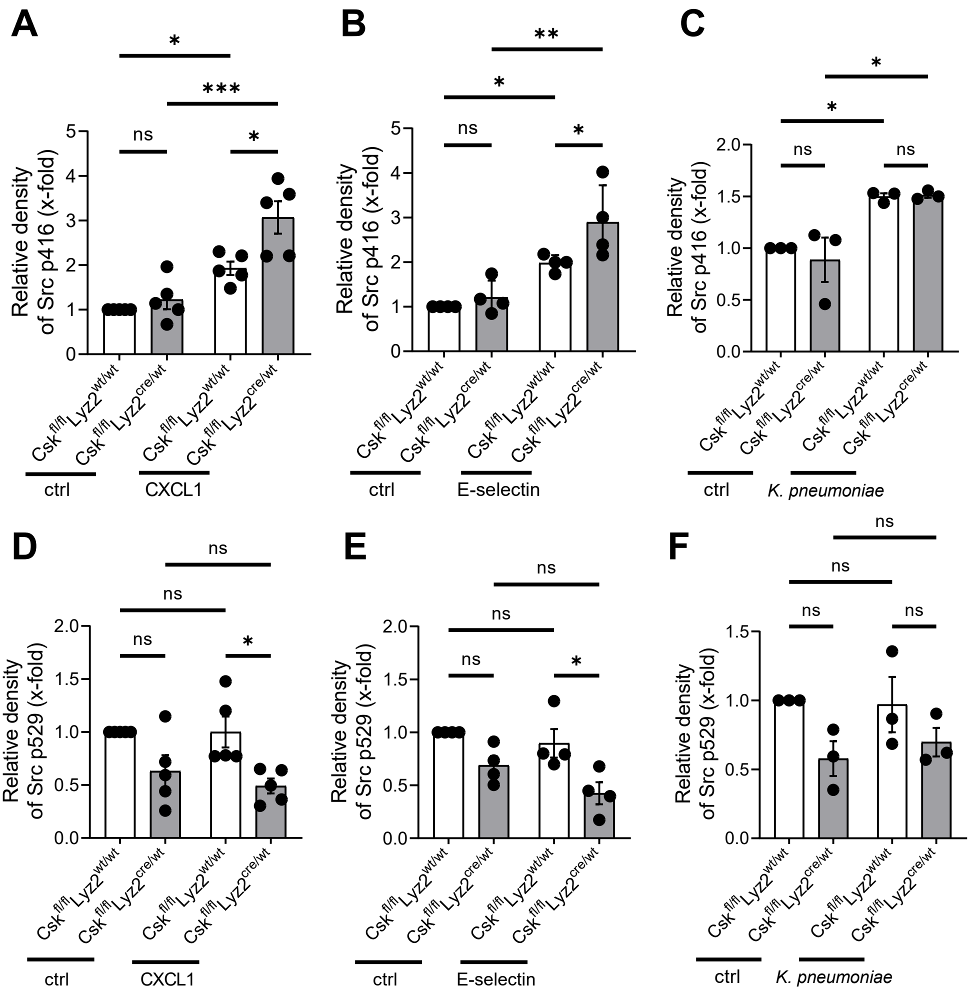

Supplemental Figure 8: Csk regulates the activity of Src kinases after CXCL1 and E-selectin, but not after *K. pneumoniae* stimulation. (**A**-**F**) Bone marrow-derived neutrophils of *Csk*$^{fl/fl}$*Lyz2*$^{wt/wt}$ and *Csk*$^{fl/fl}$*Lyz2*$^{cre/wt}$ mice were left untreated or were stimulated with CXCL1 for 1 min, E-selectin for 5 min or *K. pneumoniae* for 1 min prior lysis. Afterwards, lysates were immunoblotted with an Ab against tSrc, phospho-Src Y416 or Y529. Quantification of the phosphorylated Src Y416 (A-C) and Y529 (D-F) as a relative density of tSrc. (n as indicated, mean ± SEM, 1-way-ANOVA, *p<0.05; **p<0.01; ***p<0.001).

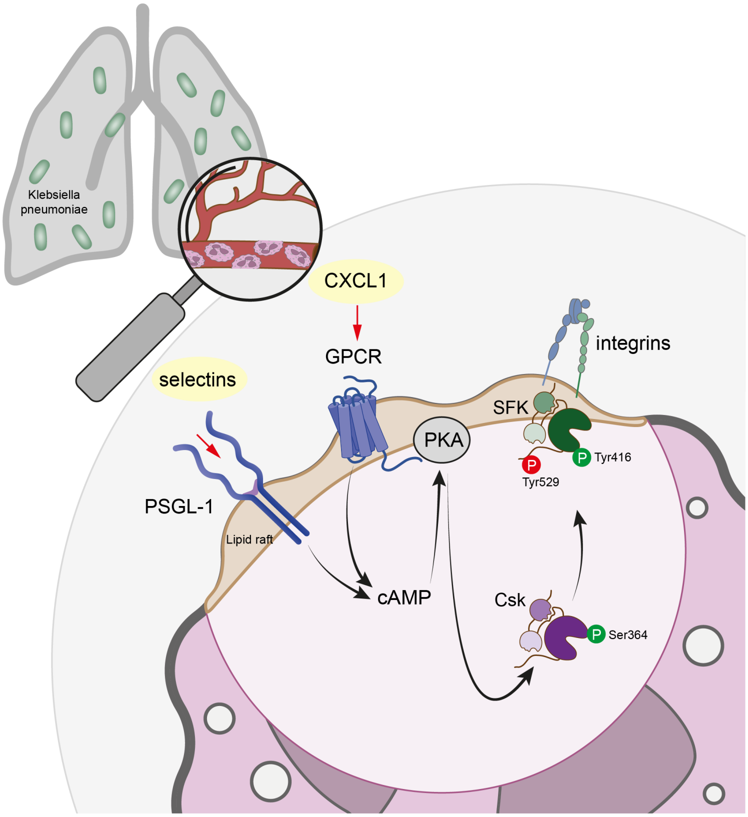

Supplemental Figure 9: Csk dampens neutrophil adhesion and recruitment in *Klebsiella pneumoniae* infected lungs. Selectin binding to PSGL-1 and CXCL1 binding to G protein-coupled receptors (GPCRs) on the neutrophil surface lead to increased intracellular cAMP levels and initiate a regulatory signaling cascade. Protein kinase A (PKA) modulates the activation status of Csk and Csk subsequently phosphorylates Src family kinases (SFKs). This pathway modulates integrin activation and neutrophil functionality.

SUPPLEMENTAL METHODS

Sysmex analysis

White blood cell, neutrophil and monocyte count were determined by an Automated Hematology Analyzer (Sysmex XN-1000^{TM}).

Analysis of steady state immune cells

Mice were anaesthetized by intraperitoneal injection of ketamine (125 mg/kg, WDT) and xylazine (12.5 mg/kg, Elanco). Lungs were lavaged four times with 0.7 mL physiological saline solution and organs were harvested as indicated. Cells in the lung and BALF were analyzed by flow cytometry (FACSCanto II, BD Biosciences). CFUs in the BALF, lungs, blood and spleen were counted by serial plating on Tryptic Soy agar plates (92).

Neutrophil isolation

Bone marrow derived neutrophils were isolated from femurs and tibias of mice using either a 1-layer or 2-layer gradient, achieving high purity and viability, as previously described by our group (1). In short, cell suspension was seeded on a 1-layer gradient of 62% Percoll (6mL, Sigma-Aldrich) or a 2-layer gradient (4 mL of Pancoll 1,077 g/mL and 4 mL of Pancoll 1,119 g/ml, Pan) and centrifuged (1-layer gradient: 1.500 g for 30 min; 2-layer gradient: 790 g for 30 min). To isolate blood neutrophils, blood from healthy donors was collected into EDTA tubes and murine blood was collected using heparin-coated syringes. Whole blood was layered on a 2-layer gradient (4 mL of Pancoll 1,077 g/mL and 4 mL of Pancoll 1,119 g/ml, Pan) and centrifuged as described before (1).

Isolation and culture of lung epithelial cells

To isolate lung epithelial cells, a previously described protocol was used with minor modifications (2). Mice were anaesthetized by intraperitoneal injection of ketamine (125 mg/kg, WDT) and xylazine (12.5 mg/kg, Elanco). Lungs were perfused with 10 mL PBS. 1 mL dispase was injected followed by 0.5 mL melted 1% agarose (Promega) through the trachea using a

23 cannula. Lungs were removed and incubated in 0.5 mL dispase for 45 min at 25°C. Afterwards, lungs were dissected and filtered through 40 µm and 20 µm cell strainers. Cell pellets were lysed in RBC Buffer (+10 µL DNaseI, Sigma). Cells were incubated with biotinylated CD45 antibody (BD Biosciences) and biotinylated CD16/32 antibody (BD Biosciences) and were purified by using MagneSpheres (Promega). Lung epithelial cells were plated on fibronectin (Sigma) coated 12-well dish in airway epithelial cell growth media (Promocell) for 5 days.

qPCR of lung epithelial cells

RNA extraction was performed using RNAeasy Mini Kit (Qiagen). RNA concentration and purity were measured on a Nanodrop2000 (Thermo Fisher Scientific). A RevertAid First Strand cDNA Synthesis Kit (Thermo Fisher Scientific) was applied for cDNA synthesis. Reaction was carried out in a vapo.protect thermocycler (Eppendorf) and a 7900HT Fast-Real Time PCR System (Thermo Fisher Scientific). Beta-Actin was used as reference gene in all samples. Primers were from Qiagen.

Imaging of NET formation by H3Cit staining

Imaging of NET formation was performed as described elsewhere (1). Murine neutrophils were purified using a 1-layer gradient. Neutrophils were placed on a 0.01% poly-L-lysine coated 8-well µ-slide (Ibidi) and incubated with *K. pneumoniae* (MOI 50) for 3 h (37 °C, 5% CO_2). Cells were then fixed with 4% PFA followed by incubation in blocking solution (2% BSA, 0.2% Triton X-100 in PBS) for 30 min. Slides were stained with a 1:300 anti-histone H3 (citrulline R2 + R8 + R17) antibody (Abcam) for 1 h, followed by a 1:200 secondary antibody for 30 min (goat anti-rabbit IgG, Thermo Fisher Scientific). Neutrophils were stained with DAPI (0.2 µg/mL, 15 min, D9542, Sigma-Aldrich). Imaging of NET formation was performed using a Lionheart FX Automated Microscope (BioTek Instruments).

Cell lines and constructs

Stable knockdown of CSK in promyelocytic HL-60 cells (Sigma-Aldrich) was performed by lentiviral transduction of short hairpin RNA (shRNA) as described previously (sequence: CGAGGAGGTGTACTTTGAGAA) (3). The knockdown efficiency was confirmed by Western Blot using antibodies against Csk (C74C1, Cell Signaling). During cell culture, the Csk knockdown was maintained by puromycin selection.

References

1. Hellenthal KEM, et al. Glutamine modulates neutrophil recruitment and effector functions during sterile inflammation. *J Leukoc Biol*. 2024. doi:10.1093/jleuko/qiae243
2. Chen Q, Liu Y. Isolation and culture of mouse alveolar type II cells to study type II to type I cell differentiation. *STAR Protoc*. 2021;2(1):100241. doi:10.1016/j.xpro.2020.100241
3. Herter JM, et al. Integrin activation by P-Rex1 is required for selectin-mediated slow leukocyte rolling and intravascular crawling. *Blood*. 2013;121(12):2301-2310. doi:10.1182/blood-2012-09-457085
