# Supplementary material for: Csk-mediated Src family kinase regulation dampens neutrophil infiltration during pulmonary infection
Source: JCI Insight. 2025 Jun 10;10(14):e188323. doi: 10.1172/jci.insight.188323 (PMC12288981; doi:10.1172/jci.insight.188323)

Full unedited blot for
Figure 6A & B

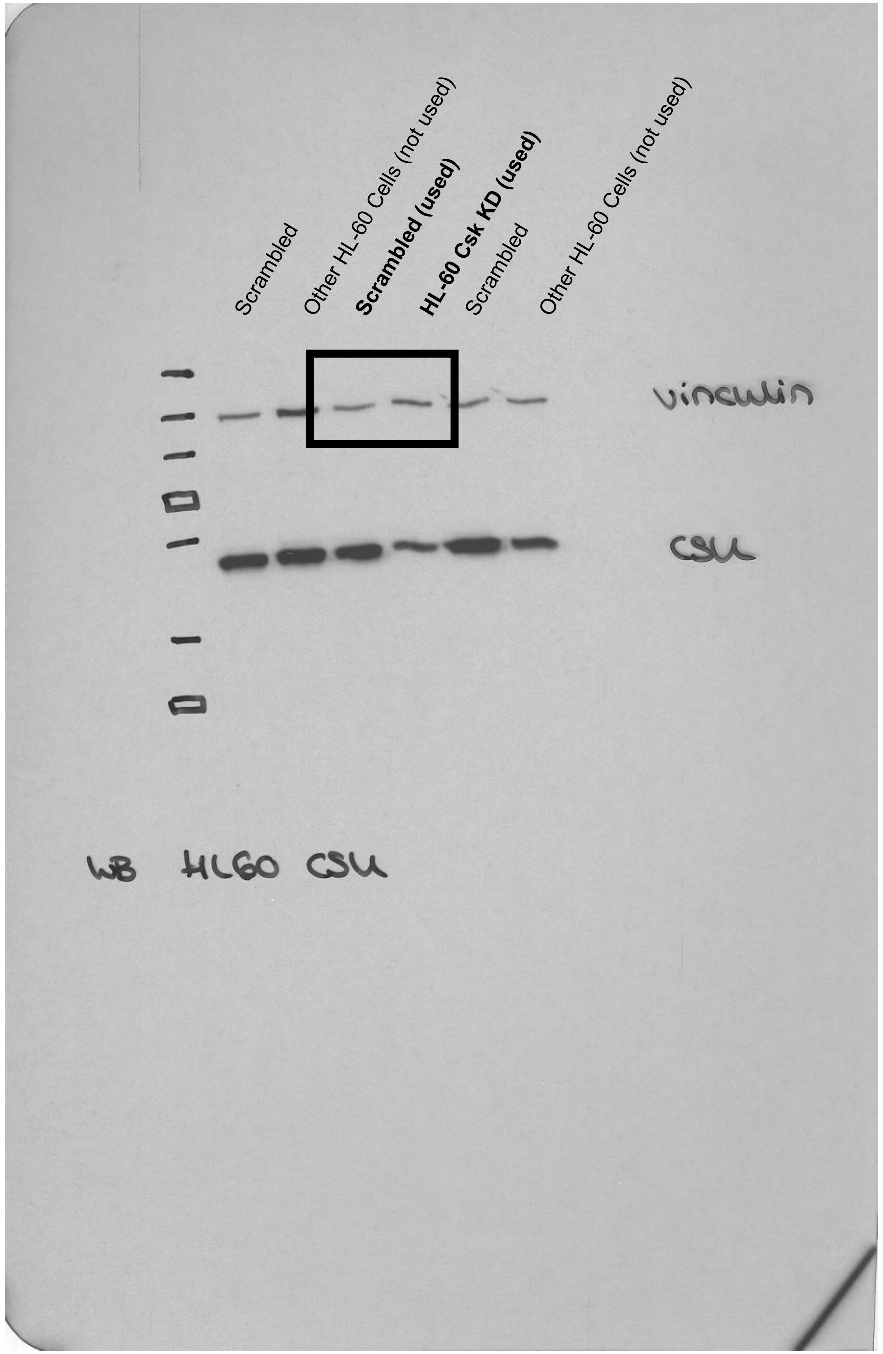

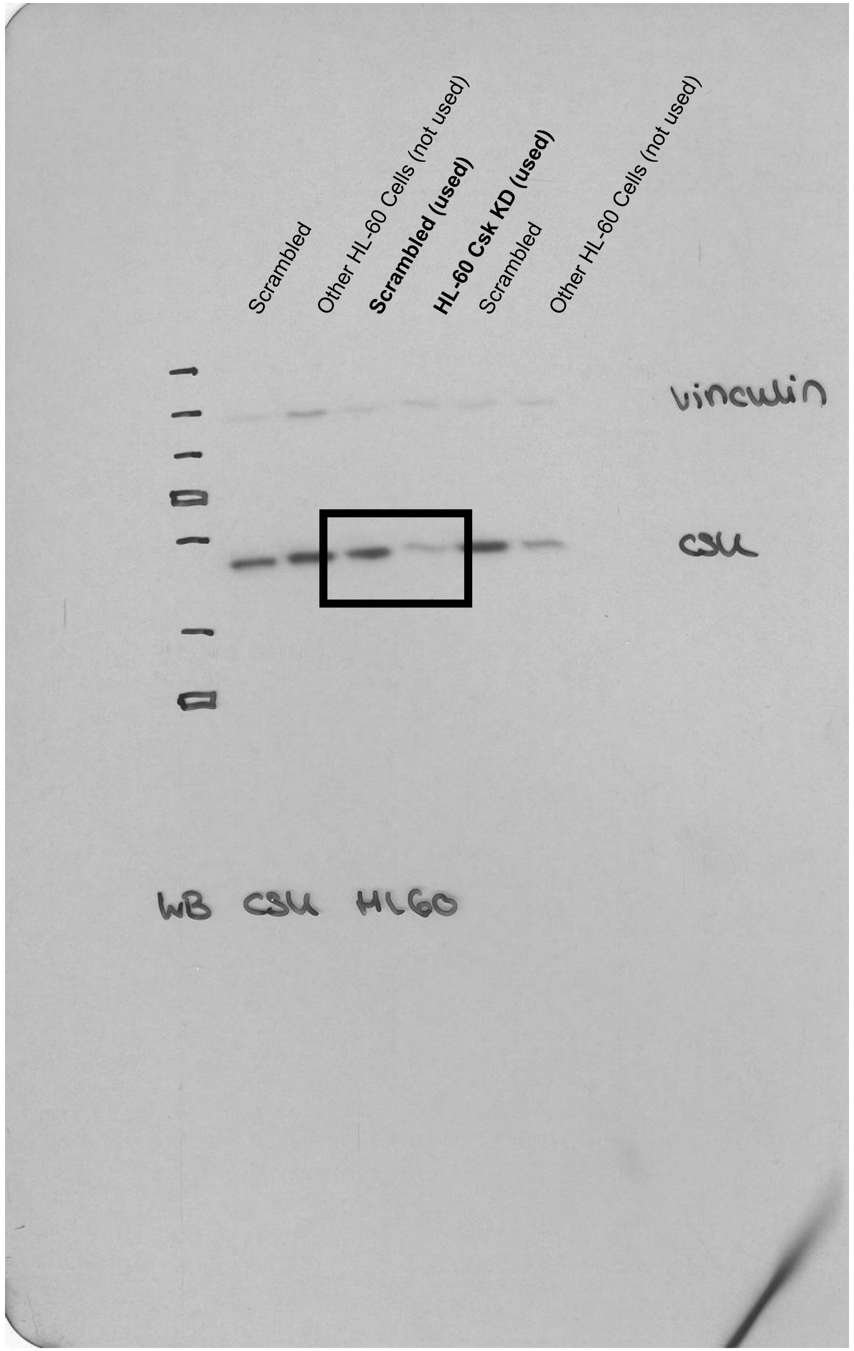

Full unedited blot for
Figure 6A & B

Scrambled (used)
HL-60 Csk KD (used)
HL-60 Csk KD (used)
Scrambled
Other HL-60 Cells (not used)
Other HL-60 Cells (not used)

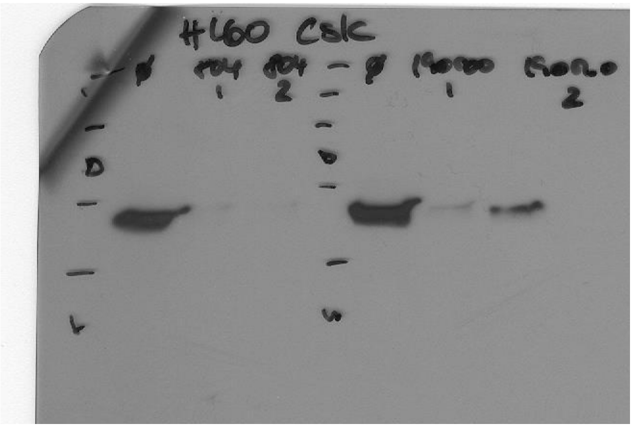

Scrambled (used)
HL-60 Csk KD (used)
HL-60 Csk KD (used)
Scrambled
Other HL-60 Cells (not used)
Other HL-60 Cells (not used)

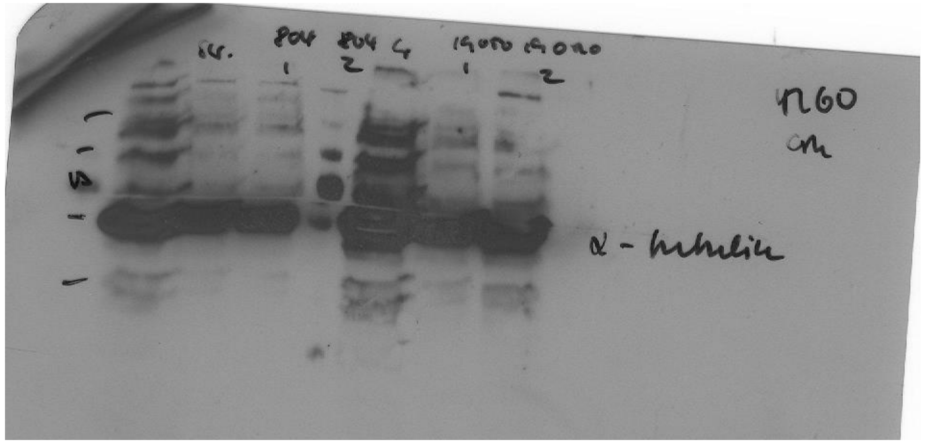

Full unedited blot for
Figure 7 E &
Supplemental Figure 8 A & D

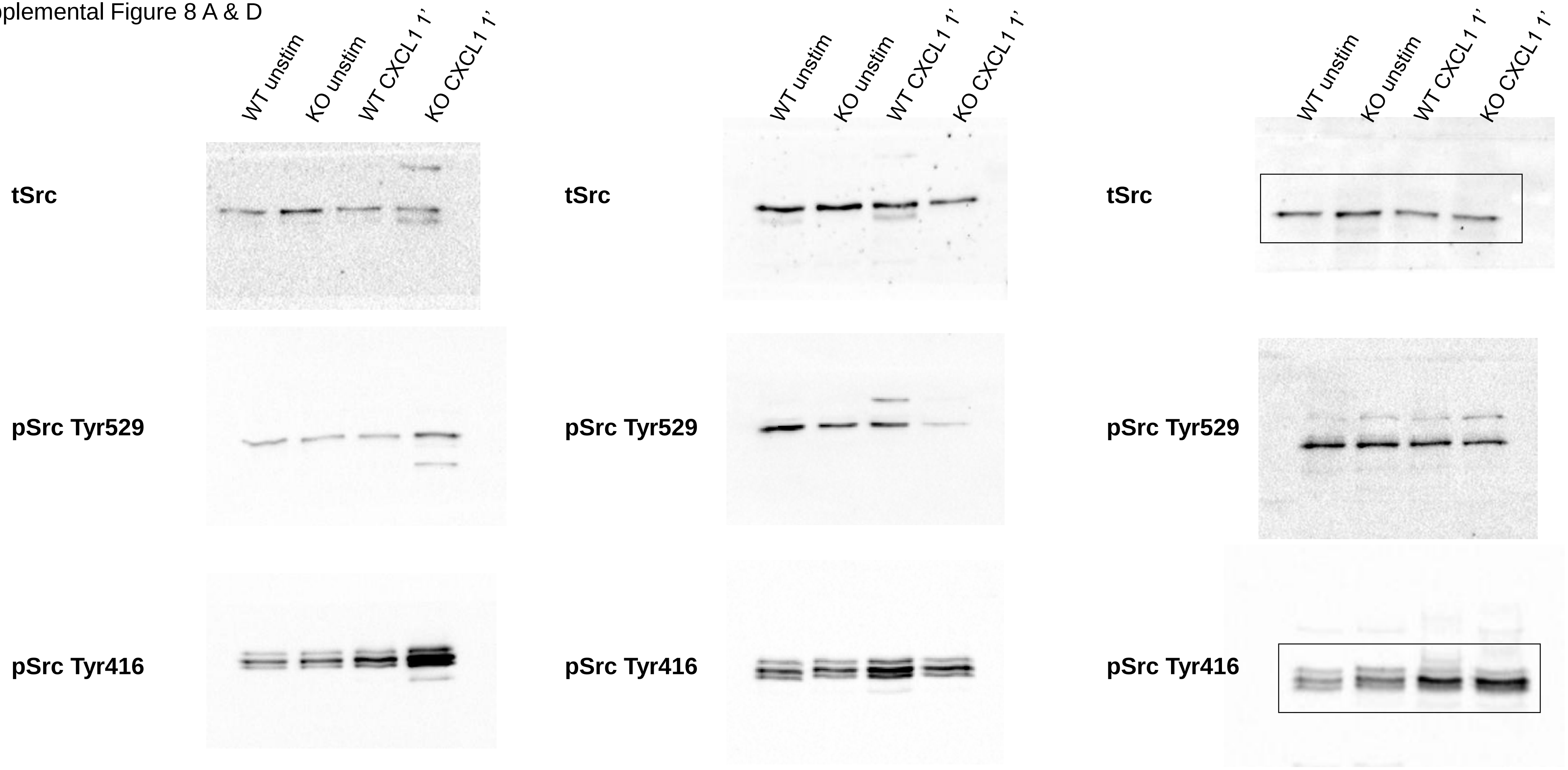

Full unedited blot for
Figure 7 E &
Supplemental Figure 8 A & D

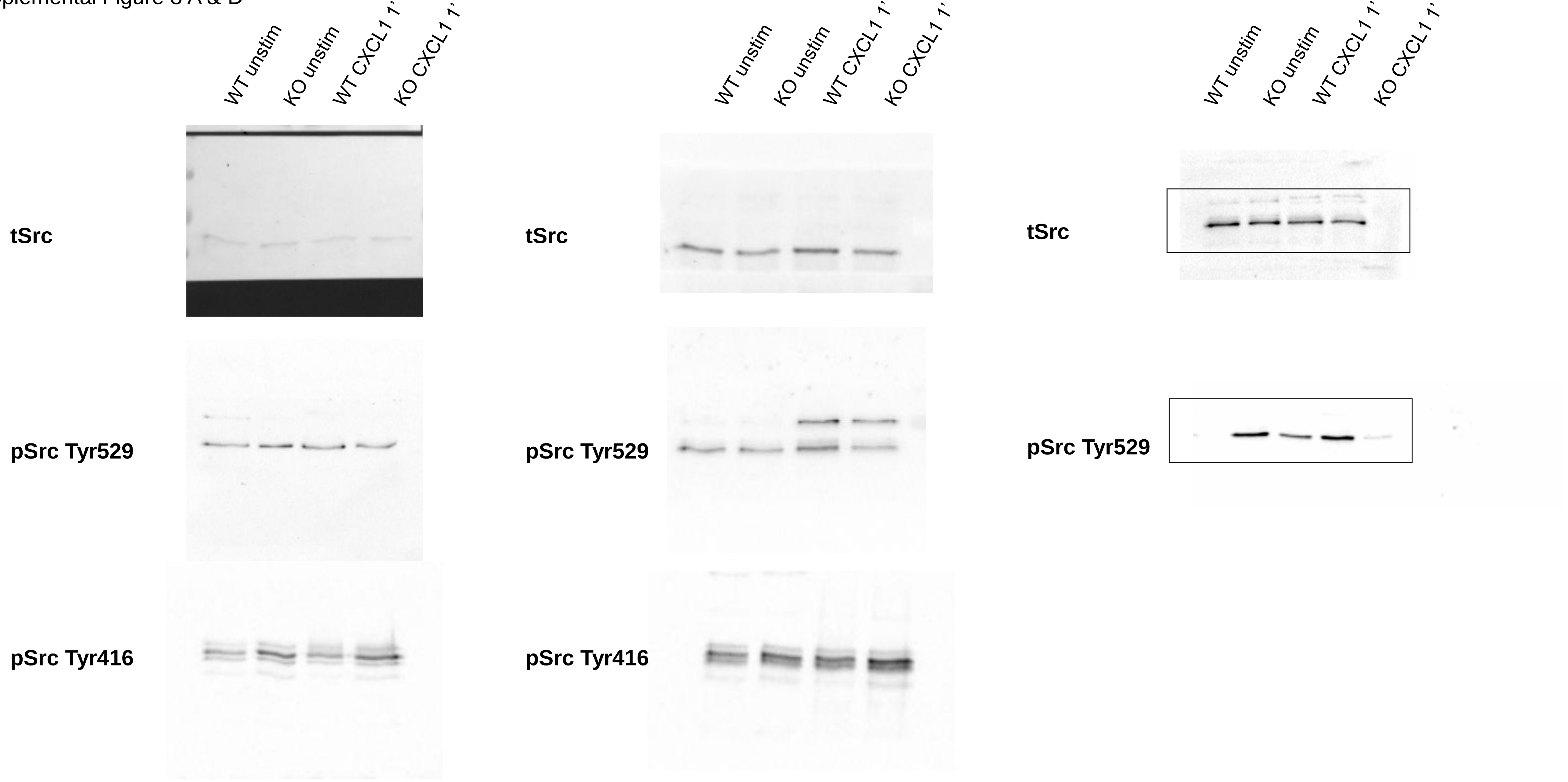

Full unedited blot for
Figure 7 F &
Supplemental Figure 8 B & E

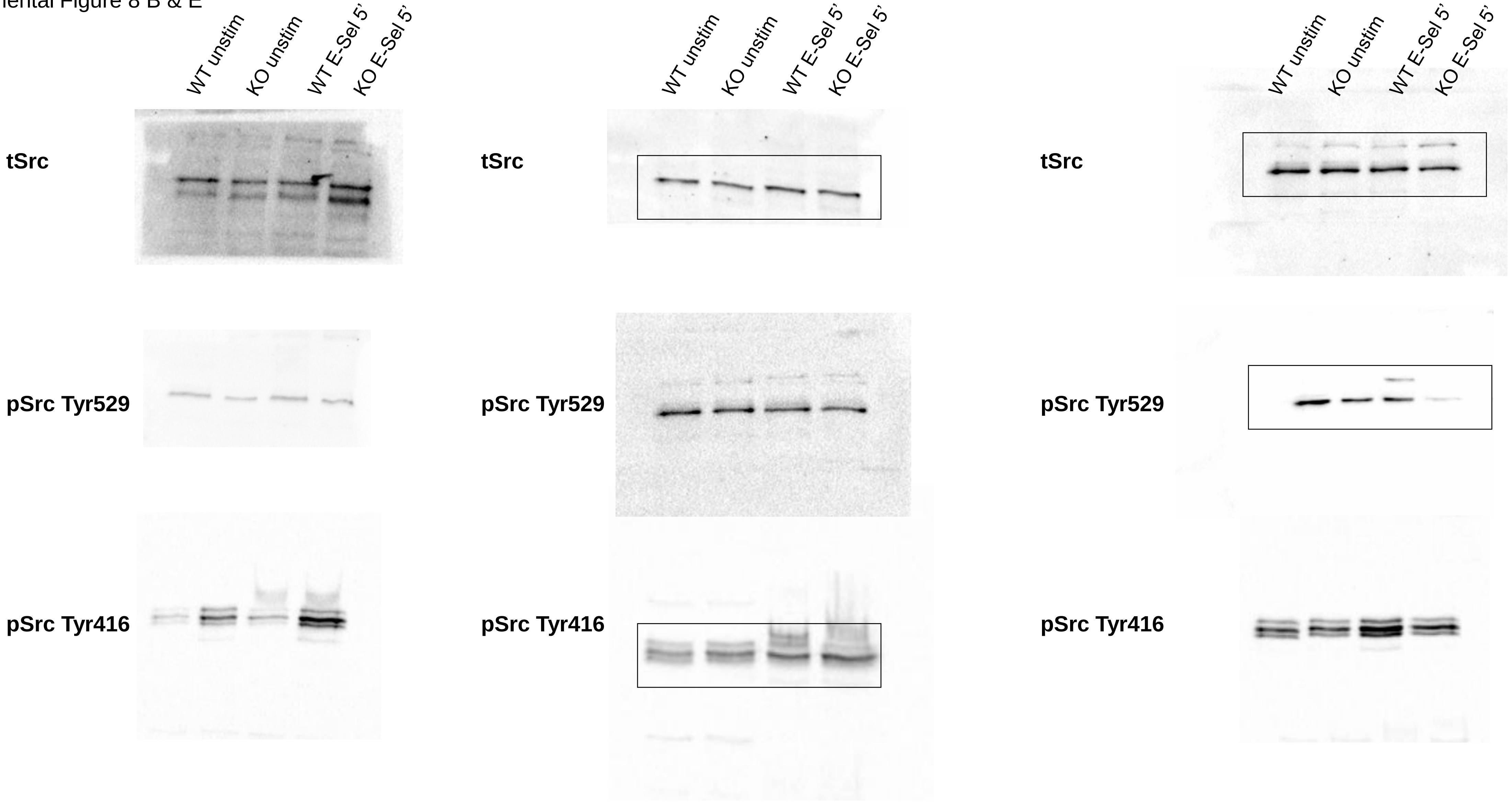

Full unedited blot for
Figure 7 F &
Supplemental Figure 8 B & E

WT / KO mixed up when applied to gel

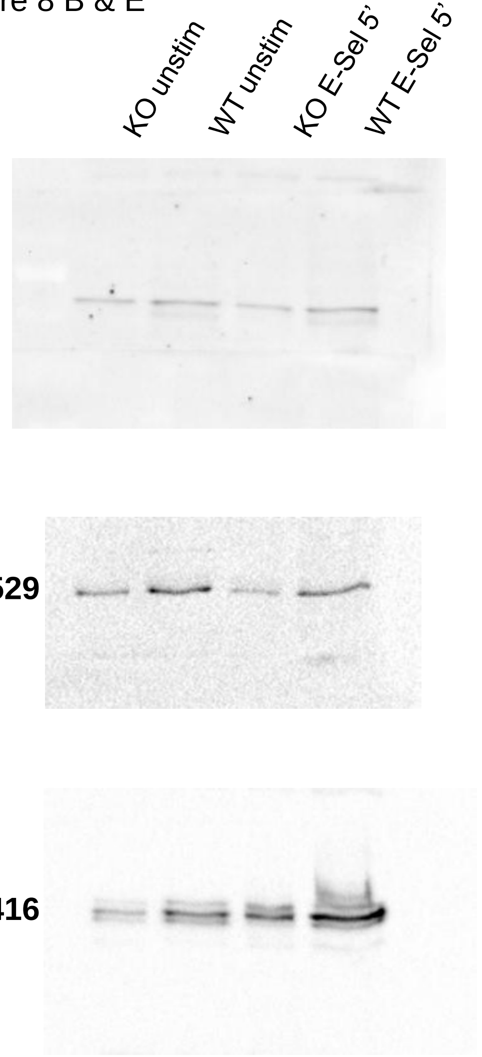

Full unedited blot for
Figure 7 G &
Supplemental Figure 8 C & F

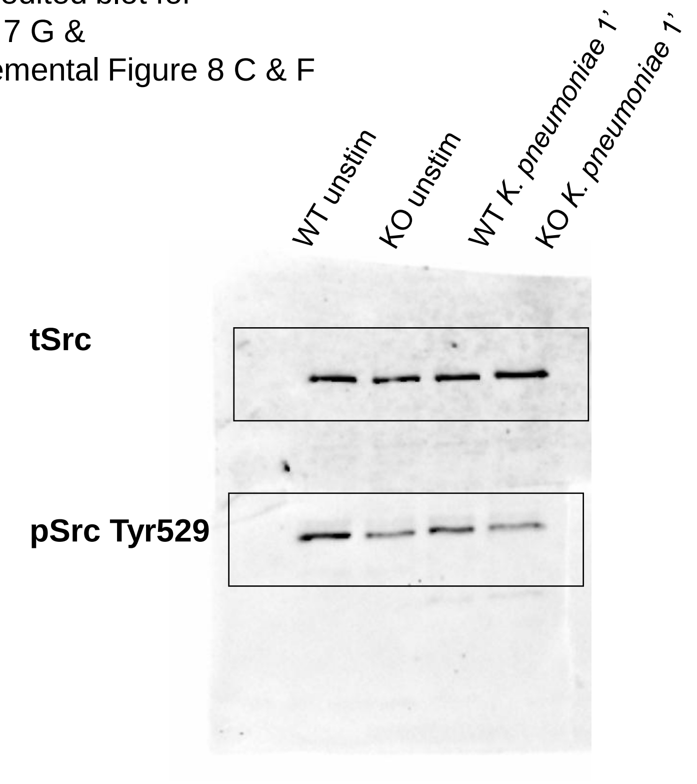

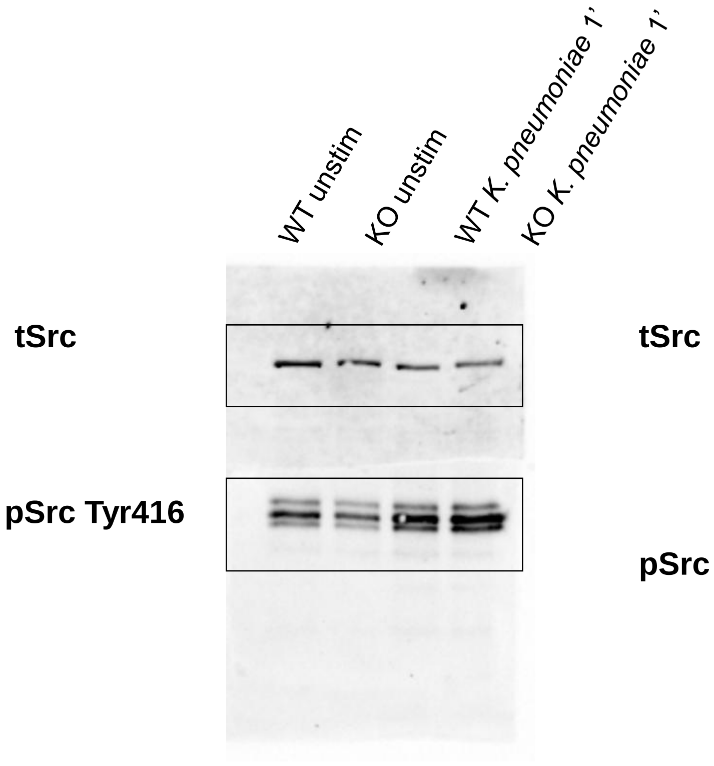

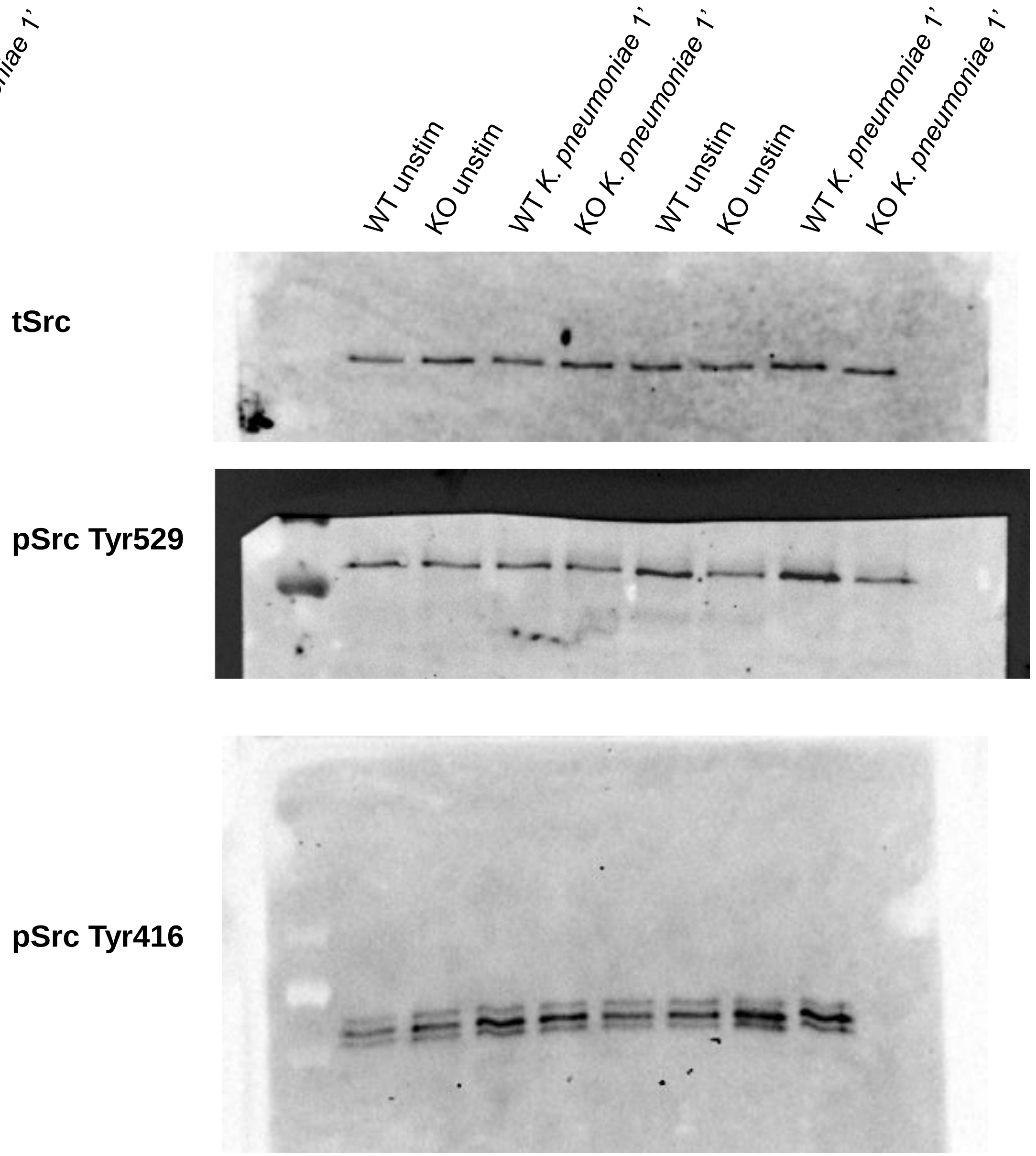

Full unedited blot for
Supplemental Figure 2 E

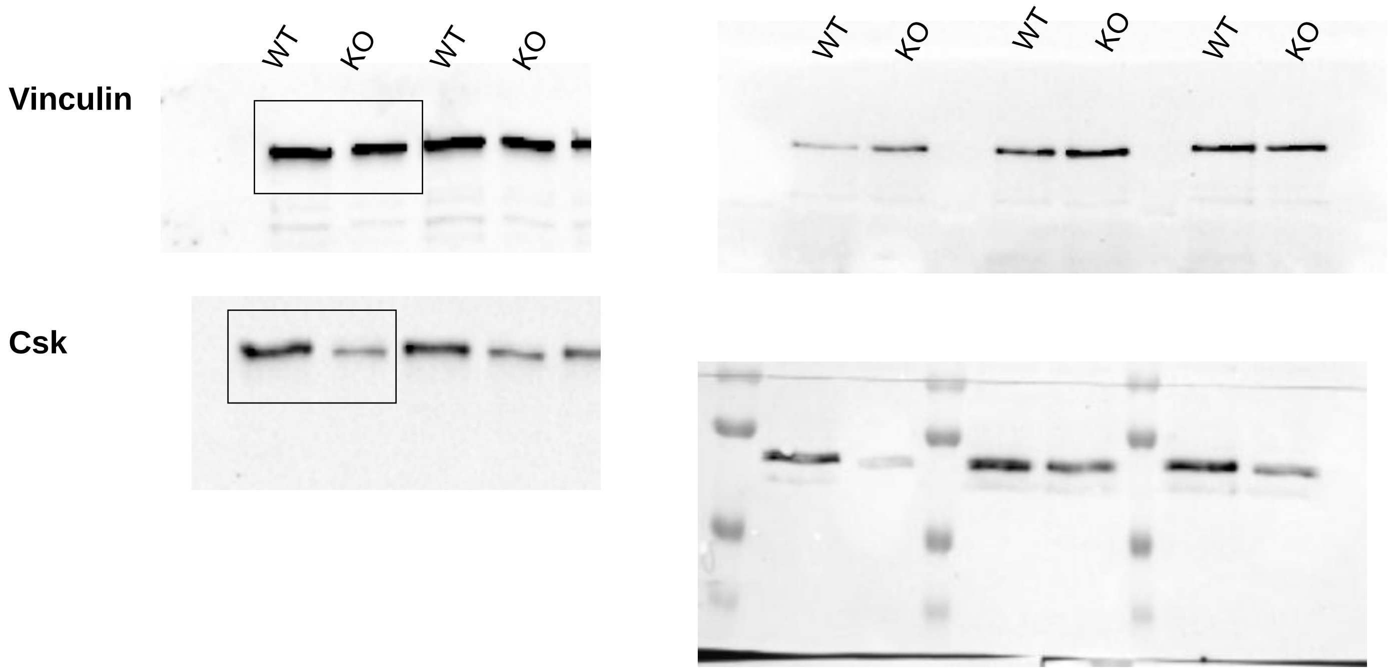

Full unedited blot for
Supplemental Figure 2 F

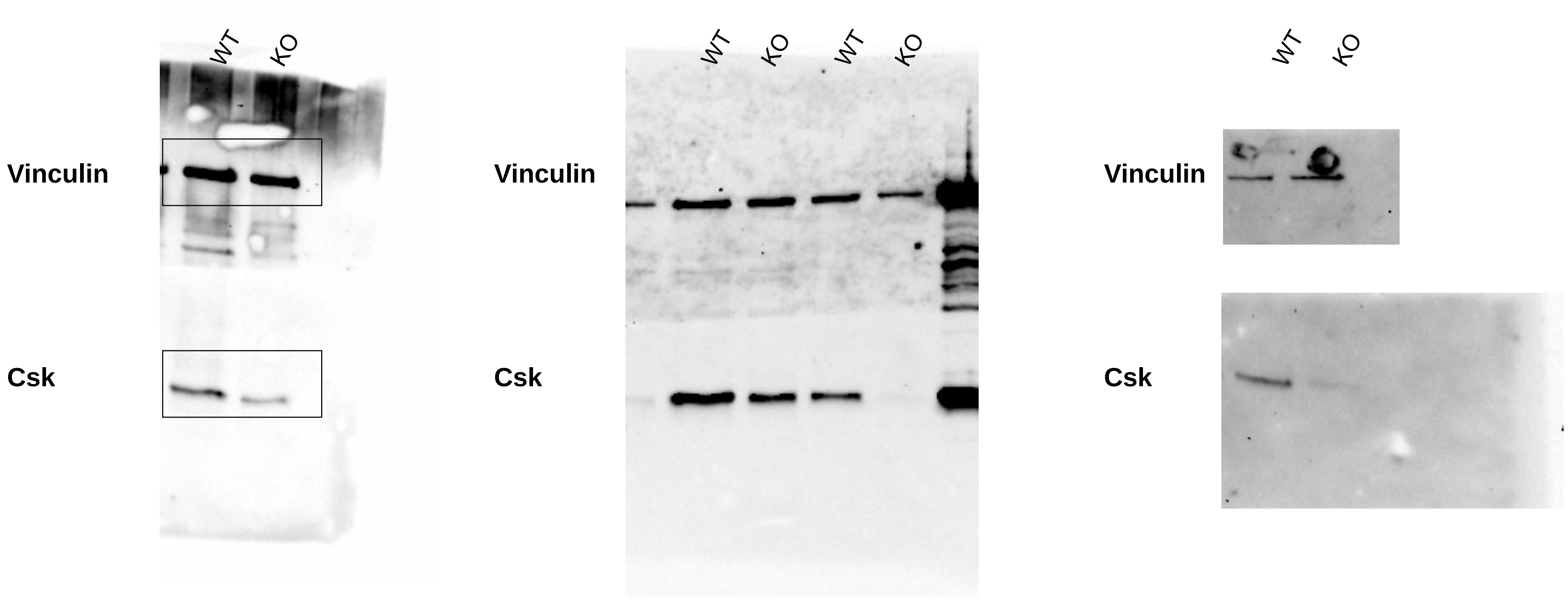

Full unedited blot for
Supplemental Figure 2 G

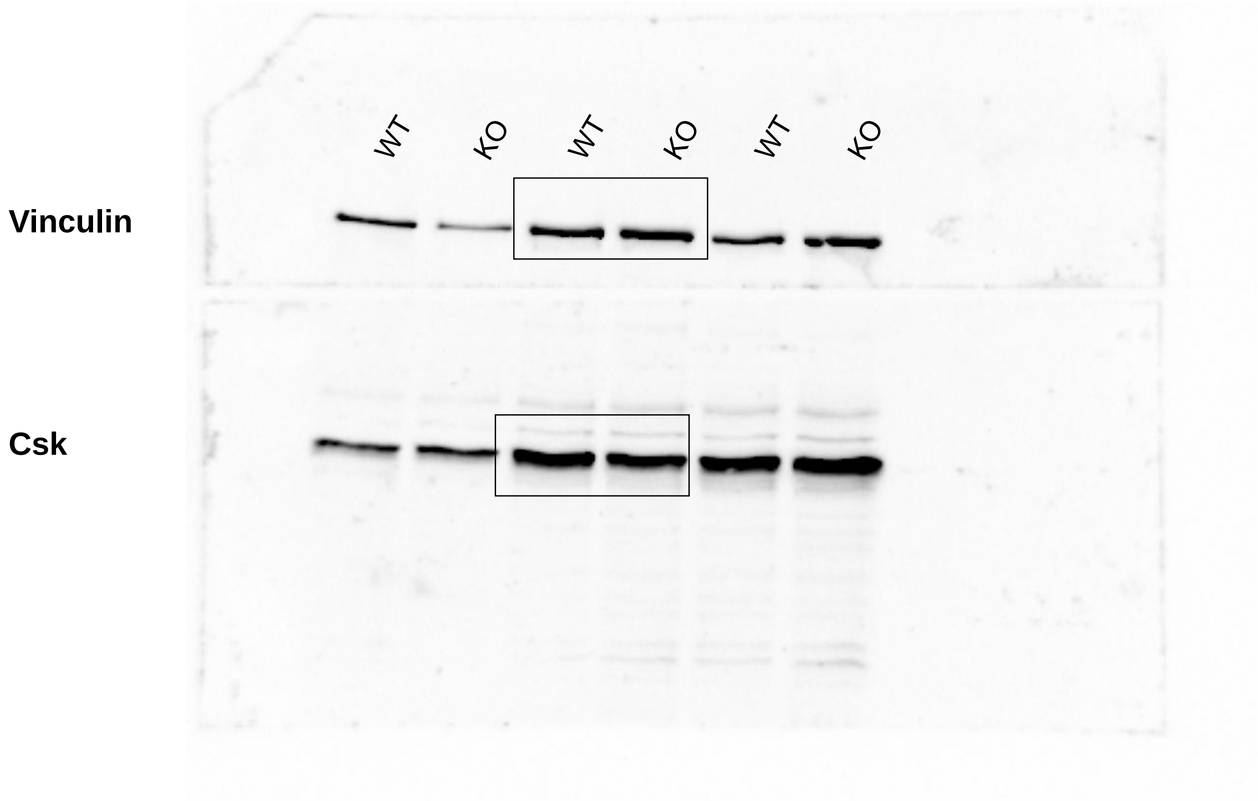

Full unedited blot for
Supplemental Figure 2 H

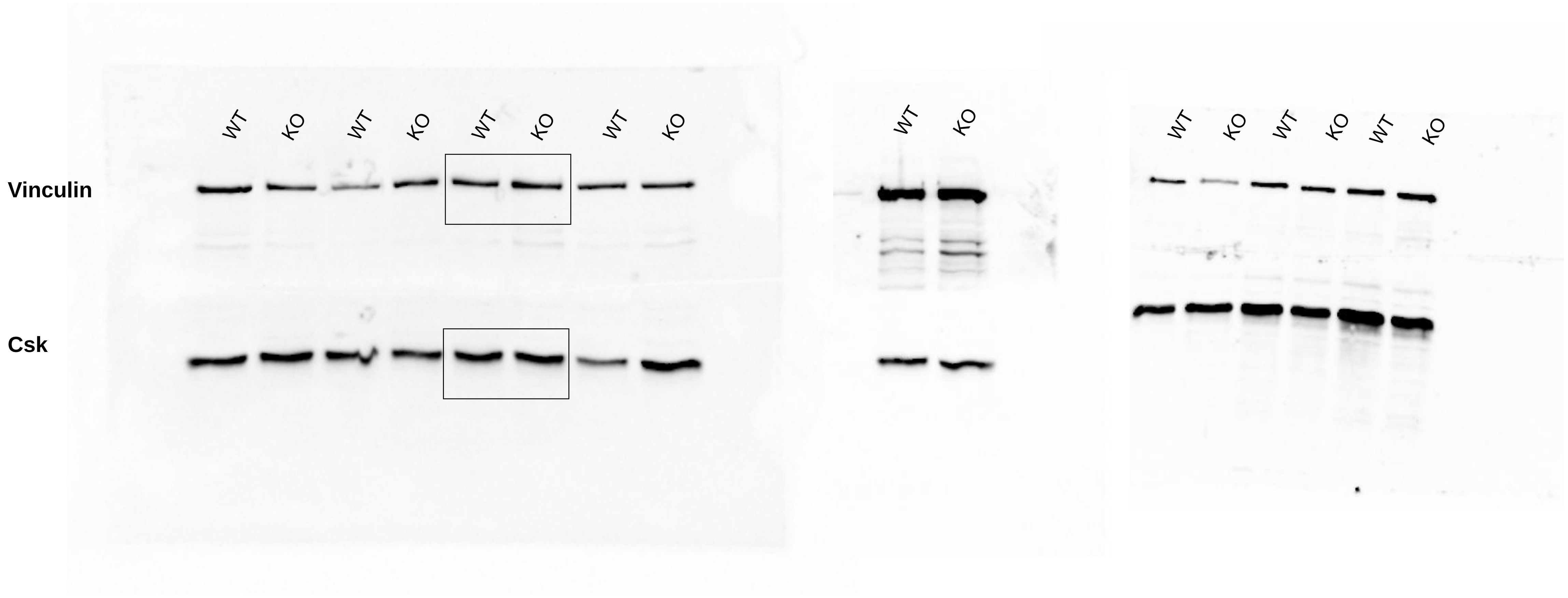

Supplement: Unedited blot and gel images [file jciinsight-10-188323-s030.pdf]
